# Genetically Predicted Midlife Blood Pressure and Coronary Artery Disease Risk: Mendelian Randomization Analysis

**DOI:** 10.1161/JAHA.120.016773

**Published:** 2020-07-04

**Authors:** Dipender Gill, Marios K. Georgakis, Verena Zuber, Ville Karhunen, Stephen Burgess, Rainer Malik, Martin Dichgans

**Affiliations:** ^1^ Department of Epidemiology and Biostatistics School of Public Health Imperial College London London United Kingdom; ^2^ Institute for Stroke and Dementia Research University Hospital of Ludwig‐Maximilians‐University Munich Germany; ^3^ Medical Research Council Biostatistics Unit Cambridge Institute of Public Health Cambridge United Kingdom; ^4^ Cardiovascular Epidemiology Unit Department of Public Health and Primary Care University of Cambridge United Kingdom; ^5^ Munich Cluster for Systems Neurology Munich Germany; ^6^ German Centre for Neurodegenerative Diseases Munich Germany

**Keywords:** age, blood pressure, coronary artery disease, mendelian randomization, Cardiovascular Disease, Epidemiology, Risk Factors, Hypertension

## Abstract

**Background:**

Elevated blood pressure is a major cause of cardiovascular morbidity and mortality. However, it is not known whether midlife blood pressure affects later life cardiovascular risk independent of later life blood pressure.

**Methods and Results:**

Using genetic association estimates from the UK Biobank and CARDIoGRAMplusC4D consortium, univariable mendelian randomization was performed to investigate the total effect of genetically predicted mean arterial pressure (MAP) at age ≤55 years on coronary artery disease (CAD) risk, and multivariable mendelian randomization was performed to investigate the effect of genetically predicted MAP on CAD risk after adjusting for genetically predicted MAP at age >55 years. In both univariable and multivariable mendelian randomization analyses, there was consistent evidence of higher genetically predicted MAP at age ≤55 years increasing CAD risk. This association persisted after adjusting for genetically predicted MAP at age >55 years, when considering nonoverlapping populations for the derivation of MAP and CAD risk genetic association estimates, when investigating only incident CAD events after age >55 years, and when restricting the analysis to variants with most heterogeneity in their associations with MAP ≤55 and >55 years. For a 10–mm Hg increase in genetically predicted MAP at age ≤55 years, the odds ratio of later life CAD was 1.43 (95% CI, 1.16–1.77; *P*=0.001) after adjusting for genetically predicted MAP at age >55 years.

**Conclusions:**

These mendelian randomization findings support a cumulative lifetime effect of elevated blood pressure on increasing CAD risk. Clinical and public health efforts toward cardiovascular disease reduction should optimize blood pressure control throughout life.

Elevated blood pressure is a powerful predictor of cardiovascular morbidity and mortality. In international surveys, the 874 million adults estimated to have a systolic blood pressure (SBP) >140 mm Hg in 2015 accounted for 106 deaths per 100 000 people and loss of 143 million disability‐adjusted life years.^1^ Lowering blood pressure can decrease cardiovascular risk, with a 10–mm Hg reduction in SBP estimated to reduce all‐cause mortality by 13%.^2^

To optimize clinical and public health strategies toward minimizing the burden of cardiovascular disease, it is important to understand whether there is a specific period in life when elevated blood pressure increases risk, or rather whether it is that elevated blood pressure throughout life has a cumulative effect. Observational studies have shown that elevated blood pressure in midlife is associated with increased risk of cardiovascular disease in later life and represents an independent risk factor, even after adjusting for blood pressure in older age.^3^, ^4^, ^5^ However, inferring causal effects from such associations can be difficult because of the possibility of confounding, reverse causation, and measurement error. The mendelian randomization (MR) paradigm overcomes some of these limitations by using genetic variants as instrumental variables for studying the effect of an exposure on an outcome. The random allocation of genetic variants at conception means that their associations are less vulnerable to environmental confounding and reverse causation, and their cumulative lifelong effect reduces the impact of measurement error.

The aim of the current study was to generate genetic instruments for mean arterial pressure (MAP) at age ≤55 years and MAP at age >55 years, and thus investigate within the MR paradigm whether genetically predicted MAP in midlife affects risk of coronary artery disease (CAD) in later life, independent of genetically predicted MAP in later life.

## Methods

### Overall Study Design

Separate genome‐wide association study (GWAS) analyses for MAP in individuals aged ≤55 years and in individuals aged >55 years were performed in UK Biobank. These age categories were selected because they reflect the approximate transition toward increasing arterial stiffness,^6^ and also split the UK Biobank cohort approximately in half. MAP was selected as the blood pressure trait of interest because it provides an estimate of overall arterial blood pressure during a complete cardiac cycle^7^ and represents a predictor of cardiovascular risk in both younger and older adults.^8^ Instruments for MAP in individuals aged ≤55 years were applied in univariable and multivariable MR analysis to investigate their effect on CAD risk. Two models were applied for univariable MR: model 1 considered outcome genetic association estimates from CARDIoGRAMplusC4D,^9^ and model 2 considered outcome genetic association estimates based on incident CAD events at age >55 years in UK Biobank. In model 2, the UK Biobank cohort was split on the basis of participant linkage to primary care data and genetic association estimates for MAP and CAD were obtained from the different subsets to avoid potential bias related to participant overlap^10^; model 2 further served as a sensitivity analysis to explore potential bias related to the inclusion of recurrent CAD events and CAD events at age ≤55 years in the CARDIoGRAMplusC4D data used for model 1. For multivariable MR, the effect of genetically predicted MAP at age ≤55 years was adjusted for the effect of genetically predicted MAP at age >55 years when investigating effects on CAD risk. A further third model was also applied in the multivariable MR setting, which only included the instrument variants from model 1 that demonstrated heterogeneity between their associations with MAP in those aged ≤55 and >55 years outside the interdecile range of the distribution expected under the null hypothesis of homogeneity. Model 3 was performed as a sensitivity analysis to explore the potential impact of weak instrument bias in the multivariable MR setting, particularly as the genetic predictors of MAP in individuals aged ≤55 years may be closely related to those for individuals aged >55 years.^11^ The data sources used to obtain genetic association estimates in the different analysis models are summarized in the Table. Baseline characteristics for the UK Biobank participants used in the GWAS analyses for MAP and CAD are detailed in Table S1.

### MAP GWAS

MAP was calculated using the mean SBP and diastolic blood pressure readings (1/3×mean SBP+2/3×mean diastolic blood pressure) obtained at baseline assessment in UK Biobank, after correcting for antihypertensive medication use by adding 15 mm Hg to SBP and 10 mm Hg to diastolic blood pressure for individuals who self‐reported use of any antihypertensive medication.^12^ Only white British participants were included in GWAS analyses, and exclusions were made for up to second‐degree related individuals (relatedness coefficient <0.0884). For the MAP GWAS used to obtain genetic association estimates in model 2, we limited participants to those not included in the UK Biobank primary care data set. After dichotomization on age (≤55 and >55 years), all MAP GWAS analyses were performed using linear regression, with age, sex, principal components 1 to 20, genotyping chip, and assessment center included as covariates. The final sample sizes for the analyses used in model 1 were as follows: ≤55 years: N=162 967; and >55 years: N=245 261. The final sample sizes in model 2 were as follows: ≤55 years: N=131 435; and >55 years: N=131 584.

### Instrument Selection

For univariable MR, instruments for MAP at age ≤55 years were selected as single‐nucleotide polymorphisms that associated with MAP in individuals aged ≤55 years at genome‐wide significance (*P*<5×10^−8^) and were in pair‐wise linkage disequilibrium (*r* ^2^<0.001). For models 1 and 2 of the multivariable MR, instruments were selected as single‐nucleotide polymorphisms related at genome‐wide significance to MAP at age ≤55 years or to MAP at age >55 years in the GWAS analyses considering the whole UK Biobank cohort, after clumping to pairwise linkage disequilibrium (*r* ^2^<0.001) on the basis of the lowest *P* value for association with either trait. All clumping was performed using the TwoSampleMR package in R.^13^ In model 3 of the multivariable MR, only variants that had heterogeneity between their associations with MAP in those ≤55 and >55 years outside the interdecile range of the distribution expected under the null hypothesis of homogeneity were included.

### Outcome Genetic Association Estimates

Genetic association estimates for CAD that were used in models 1 and 3 were obtained from the CARDIoGRAMplusC4D Consortium 1000G multiethnic GWAS (77% European ancestry) of 60 801 cases and 123 504 controls.^9^

Genetic association estimates for CAD used in model 2 were obtained from UK Biobank participants with linked primary care data. These CAD diagnoses were derived from multiple sources: death records (*International Classification of Diseases, Tenth Revision* [*ICD‐10*]), hospital records (*ICD‐10* and Office of Population Censuses and Surveys‐4), and primary care data (release readV2 and readV3). We used the following codes: *ICD‐10* I20 to I25; and Office of Population Censuses and Surveys‐4 K40 to K46, K49, K50, and K75. ReadV2 and readV3 codes were extracted using a mapping from *ICD‐10* codes provided by the UK Biobank (resource 592). We retained only incident events recorded after inclusion in the UK Biobank. Only white British participants aged >55 years were included in GWAS analyses, and exclusions were made for up to second‐degree related individuals (relatedness coefficient <0.0884). GWAS analysis was performed using logistic regression, with age, sex, principal components 1 to 20, genotyping chip, and assessment center included as covariates. The final sample size was 8788 cases and 184 201 controls.

### Statistical Analysis

#### Univariable MR

Multiplicative random‐effects inverse‐variance–weighted MR was used as the main analysis for estimating the effect of genetically predicted MAP in individuals aged ≤55 years on CAD risk in the univariable setting.^14^ Contamination‐mixture method and weighted median MR were further incorporated as sensitivity analyses to explore the robustness of the findings to potential pleiotropic variants^14^ (Data S1). The MendelianRandomization package in R was used for performing inverse‐variance–weighted, contamination‐mixture, and weighted median MR.^14^

#### Multivariable MR

To estimate the effect of genetically predicted MAP at age ≤55 years on CAD risk independent of genetically predicted MAP at age >55 years, summary data multivariable MR was performed.^11^ Specifically, the CAD risk association estimates for each instrument were regressed on the association estimates for MAP in individuals aged ≤55 and >55 years, weighted for the precision of the CAD risk estimates and with the intercept fixed at 0.

### Ethical Approval and Data Availability

The data used in these analyses are publicly available. The UK Biobank study was approved by the North West Multicentre Research Ethics Committee, and all its participants provided informed consent. The UK Biobank data were accessed through application 2532. All generated results are presented in the article and its supplement. A study protocol was not preregistered. This study was reported with consideration of the STROBE‐MR Guidelines (Data S2).

## Results

**Figure 1:**
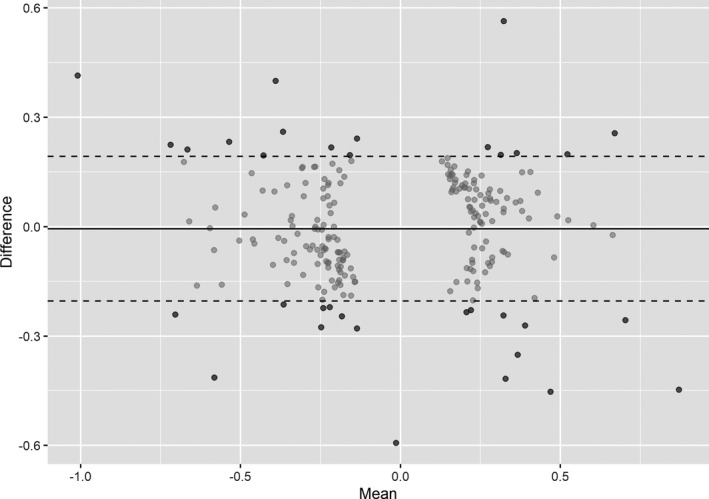
**Bland‐Altman plot depicting the heterogeneity in associations with mean arterial blood pressure (MAP) at age ≤55 and >55 years for the variants identified as having genome‐wide significant association with either trait in analyses considering the whole UK Biobank cohort.** For each variant, the *x* axis depicts the mean of the association with MAP at age ≤55 and >55 years, and the *y* axis represents the difference in association with MAP at age ≤55 and >55 years. The dashed lines depict 10th and 90th percentiles of the expected distribution of heterogeneity statistics under the null hypothesis of homogeneity (ie, the interdecile range). A total of 34 variants (colored black rather than gray) fall outside this and were used in model 3 of the multivariable mendelian randomization.

All instruments and genetic association estimates used in the MR analyses are provided in Tables S2 through S5. A Bland‐Altman plot identified 34 variants in model 1 of the multivariable MR as having heterogeneity in their associations with MAP in those aged ≤55 and >55 years outside the interdecile range of the distribution expected under the null hypothesis of homogeneity (Figure 1, Table S4), and these were applied in model 3 of the multivariable MR.

**Figure 2:**
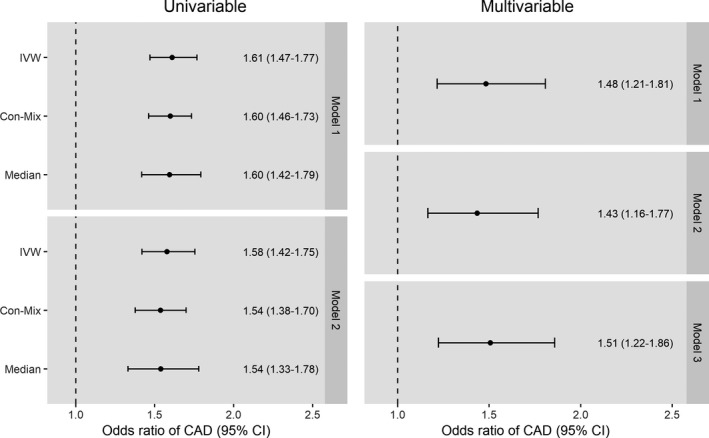
**Effect of genetically predicted mean arterial blood pressure (MAP) at age ≤55 years on risk of coronary artery disease (CAD) in univariable and multivariable mendelian randomization analyses.** All effect estimates are given per 10–mm Hg increase in MAP. Multivariable estimates are adjusted for genetically predicted MAP at age >55 years. Con‐Mix indicates contamination mixture model mendelian randomization; IVW, inverse‐variance–weighted mendelian randomization; and Median, weighted median mendelian randomization.

The univariable and multivariable MR analyses demonstrated consistent evidence of an effect of genetically predicted MAP at age ≤55 years on CAD risk across all models (Figure 2). For the univariable MR, similar results were obtained when performing the inverse‐variance–weighted, contamination‐mixture, and weighted median MR methods, which each make different assumptions about the potential inclusion of pleiotropic variants that affect CAD risk through pathways unrelated to MAP (Figure 2). Similar results were also obtained when considering CAD outcome genetic association estimates from CARDIoGRAMplusC4D or UK Biobank (Figure 2). The inverse‐variance–weighted analysis in model 2 of the univariable MR, which used nonoverlapping populations for exposure and outcome genetic association estimates and only considered incident CAD events after the age of 55 years, produced an odds ratio (OR) of 1.58 per 10–mm Hg increase in genetically predicted MAP (95% CI, 1.38–1.70; *P*<0.001).

The multivariable MR analysis, which adjusted the effect of genetically predicted MAP at age ≤55 years for genetically predicted MAP at age >55 years, produced smaller MR estimates with wider 95% CIs than the univariable MR analysis that did not make such an adjustment (Figure 2). As with the univariable MR, similar results were obtained in the various multivariable MR models considered (Figure 2). Model 2 of the multivariable MR, which adjusted for genetically predicted MAP at >55 years of age, used nonoverlapping populations for exposure and outcome genetic association estimates, and only considered incident CAD events after the age of 55 years produced an OR of 1.43 per 10–mm Hg increase in genetically predicted MAP (95% CI, 1.16–1.77; *P*=0.001).

## Discussion

This work applied the MR paradigm to generate evidence supporting an effect of midlife blood pressure on later life CAD risk independent of later life blood pressure. This finding reinforces the importance of adequate blood pressure control throughout life and is consistent with a cumulative temporal effect of elevated blood pressure on CAD risk. Our findings therefore have direct clinical and public health implications for optimizing management of blood pressure toward the goal of minimizing the burden of cardiovascular disease on both individuals and health systems.

The findings of this work build on previous conventional epidemiological research that has supported a cumulative lifetime effect of blood pressure on a range of cardiovascular outcomes, including atherosclerosis, stroke, and heart failure.^3^, ^4^, ^5^ These previous efforts have also investigated effects of blood pressure across a range of ages, including adolescence and midlife.^3^, ^4^, ^5^ Findings from these distinct populations and study designs can therefore be triangulated to generate complementary evidence supporting the case that it is cumulative and prolonged exposure to higher blood pressure that leads to the pathological processes underlying cardiovascular disease.^15^

Our study has several strengths. To our knowledge, this is the first MR study to investigate the direct effect of genetically predicted blood pressure in midlife after adjusting for genetically predicted blood pressure in older age. Compared with conventional epidemiological research, such application of the MR approach may potentially be more robust to biases related to environmental confounding, reverse causation, and measurement error. For example, by using randomly allocated genetic variants as instrumental variables for studying the effect of modifying MAP, the MR approach that we use is able to overcome confounding from factors such as smoking and lipid status. Our study also incorporates an innovative design and comprehensive range of sensitivity analyses to explore the robustness of the findings to possible violations of the underlying assumptions of the applied MR approach. Consistent evidence supporting a direct effect of midlife blood pressure on CAD risk was obtained after adjusting for genetically predicted MAP at age >55 years, when considering nonoverlapping populations for the derivation of MAP and CAD risk genetic association estimates, when investigating only incident CAD events after age >55 years, and when restricting the analysis to variants with most heterogeneity in their associations with MAP ≤55 and >55 years.

Our work also has several limitations. First, the MR paradigm measures the lifelong effect of genetic variants, and its estimates should therefore not be directly translated to assume the effect of clinical intervention on blood pressure in a given age group. Second, it was not possible to exclude the possibility that some of our analyses might have been influenced by weak instrument bias. This is particularly relevant for the multivariable MR analysis, as the associations of the genetic variants with MAP in those aged ≤55 and >55 years were closely related (Tables S4 and S5). In the univariable analyses, such bias would have been toward the null, and is therefore unlikely to be affecting our conclusions.^10^ However, in the multivariable MR settings, weak instrument bias can be either toward or away from the null.^11^ In any case, some assurance against this was provided by the consistent findings in our multivariable MR sensitivity analysis that restricted to variants with most heterogeneity in their associations with MAP in those aged ≤55 years and those aged >55 years (Figure 1), as these would be least likely to experience such weak instrument bias.^11^ Third, the use of antihypertensive medications varied between those aged ≤55 years and those aged >55 years in our GWAS analyses for MAP (Table S1). Although correction was made for antihypertensive drug use in these GWAS analyses, there may still have been some residual bias that could affect the analysis results.

In conclusion, this study uses the MR approach to generate evidence supporting an effect of midlife blood pressure on later life CAD risk that is independent of later life blood pressure. These findings build on existing conventional epidemiological research, and by considering distinct populations and analytical methods, they add to the body of science supporting that it is a cumulative effect of higher blood pressure that increases cardiovascular disease risk. Clinical and public health inventions should therefore be directed toward optimizing blood pressure control across all age groups.

## Sources of Funding

Dr Gill is supported by the Wellcome Trust 4i Programme (203928/Z/16/Z) and British Heart Foundation Centre of Research Excellence (RE/18/4/34215) at Imperial College London. Dr Georgakis is funded by a scholarship from the Onassis Foundation. Dr Burgess is supported by a Sir Henry Dale Fellowship, jointly funded by the Wellcome Trust and the Royal Society (204623/Z/16/Z). This project has received funding from the European Union's Horizon 2020 Research and Innovation Programme (666881); SVDs@target (Dr Dichgans; 667375); CoSTREAM (Dr Dichgans); the DFG as part of the Munich Cluster for Systems Neurology (SyNergy; EXC 2145 SyNergy, identifier 390857198); the CRC 1123 (B3; Dr Dichgans) and project DI 722/13‐1; the Corona Foundation (Dr Dichgans); the LMUexcellent Fond (Dr Dichgans); the e:Med Program (e:AtheroSysMed; Dr Dichgans); and the FP7/2007‐2103 European Union Project CVgenes@target (grant agreement Health‐F2‐2013‐601456; Dr Dichgans). The funding sources had no role in the design, acquisition of data, analysis, interpretation, or write up of this study.

## Disclosures

Dr Gill is employed part‐time by Novo Nordisk. The remaining authors have no disclosures to report.

**Table 1:** Data Sources Used to Obtain Genetic Association Estimates in the Univariable and Multivariable MR Analysis Models

Variable	Univariable MR		Multivariable MR		
	Model 1	Model 2	Model 1	Model 2	Model 3
MAP instruments and genetic association estimates	Individuals aged ≤55 y in the whole UK Biobank	UK Biobank participants aged ≤55 y without linked primary care data	Individuals aged ≤55 and >55 y in the whole UK Biobank	UK Biobank participants aged ≤55 y without linked primary care data	Individuals aged ≤55 and >55 y in the whole UK Biobank. Only instruments demonstrating heterogeneity between their associations with MAP in those ≤55 and >55 y outside the interdecile range of the distribution expected under the null hypothesis of homogeneity were included.
Coronary artery disease genetic association estimates	CARDIoGRAMplusC4D	UK Biobank participants aged >55 y with linked primary care data	CARDIoGRAMplusC4D	UK Biobank participants aged >55 y with linked primary care data	CARDIoGRAMplusC4D

MAP indicates mean arterial pressure; and MR, mendelian randomization.

## Supporting information


**Data S1–S2**
**Tables S1–S5**
**References** **16** **and** **17**
