# Supplementary material for: Genetically Predicted Midlife Blood Pressure and Coronary Artery Disease Risk: Mendelian Randomization Analysis
Source: J Am Heart Assoc. 2020 Jul 4;9(14):e016773. doi: 10.1161/JAHA.120.016773 (PMC7660704; doi:10.1161/JAHA.120.016773)
Supplement: Supplementary file 1 — Data S1–S2 Tables S1–S5 References 16 and 17 [file JAH3-9-e016773-s001.pdf]

SUPPLEMENTAL MATERIAL

Data S1.

Supplemental Methods

Univariable Mendelian randomization

n sensitivity analyses

Contamination-mixture method and weighted median Mendelian randomization (MR) were incorporated as sensitivity analyses in univariable MR to explore the robustness of the findings to potential pleiotropic variants (16, 17). The contamination-mixture method assumes that the MR estimates obtained from valid instruments follow a normal distribution centered on the true causal effect estimate and that those derived from invalid instruments follow a normal distribution centered on the null (16). A likelihood function is then specified and maximized for allocating each instrument variant to one of the two mixture distributions (16). The weighted median method first orders the MR estimates obtained from individual variants by their magnitude weighted for their precision (17). The median value is then selected as the overall MR estimate and standard errors are calculated by bootstrapping (17).

Data S2.

Supplemental Checklist

Research Checklist – STROBE-MR Reporting Guidelines

Davey Smith G, Davies NM, Dimou N, Egger M, Gallo V, Golub R, et al. STROBE-MR: Guidelines for strengthening the reporting of Mendelian randomization studies. https://doi.org/10.7287/peerj.preprints.27857v1. PeerJ Preprints. 2019;7:e27857v1.

1. TITLE and ABSTRACT

Indicate Mendelian randomization as the study's design in the title and/or the abstract.

Title and Abstract

INTRODUCTION

2. Background

Explain the scientific background and rationale for the reported study. Is causality between exposure and outcome plausible? Justify why MR is a helpful method to address the study question.

Introduction

3. Objectives

State specific objectives clearly, including pre-specified causal hypotheses (if any).

Abstract and Background

METHODS

4. Study design and data sources

Present key elements of study design early in the paper. Consider including a table listing sources of data for all phases of the study. For each data source contributing to the analysis, describe the following:

a) Describe the study design and the underlying population from which it was drawn.

Describe also the setting, locations, and relevant dates, including periods of recruitment, exposure, follow-up, and data collection, if available.

b) Give the eligibility criteria, and the sources and methods of selection of participants.

c) Explain how the analysed sample size was arrived at.

d) Describe measurement, quality and selection of genetic variants.

e) For each exposure, outcome and other relevant variables, describe methods of assessment and, in the case of diseases, the diagnostic criteria used.

f) Provide details of ethics committee approval and participant informed consent, if relevant.

Methods

5. Assumptions

Explicitly state assumptions for the main analysis (e.g. relevance, exclusion, independence, homogeneity) as well assumptions for any additional or sensitivity analysis.

Methods

6. Statistical methods: main analysis

Describe statistical methods and statistics used.

a) Describe how quantitative variables were handled in the analyses (i.e., scale, units, model).

b) Describe the process for identifying genetic variants and weights to be included in the analyses (i.e, independence and model). Consider a flow diagram.

c) Describe the MR estimator, e.g. two-stage least squares, Wald ratio, and related statistics.

Detail the included covariates and, in case of two-sample MR, whether the same covariate set was used for adjustment in the two samples.

d) Explain how missing data were addressed.

e) If applicable, say how multiple testing was dealt with.

Methods

7. Assessment of assumptions

Describe any methods used to assess the assumptions or justify their validity.

Methods and Discussion

8. Sensitivity analyses

Describe any sensitivity analyses or additional analyses performed.

Methods

9. Software and pre-registration

a) Name statistical software and package(s), including version and settings used.

Methods

b) State whether the study protocol and details were pre-registered (as well as when and where).

Methods

RESULTS

10. Descriptive data

a) Report the numbers of individuals at each stage of included studies and reasons for exclusion. Consider use of a flow-diagram.

b) Report summary statistics for phenotypic exposure(s), outcome(s) and other relevant variables (e.g. means, standard deviations, proportions).

c) If the data sources include meta-analyses of previous studies, provide the number of studies, their reported ancestry, if available, and assessments of heterogeneity across these studies. Consider using a supplementary table for each data source.

d) For two-sample Mendelian randomization:

i. Provide information on the similarity of the genetic variant-exposure associations between the exposure and outcome samples.

ii. Provide information on extent of sample overlap between the exposure and outcome data sources.

Methods, Results and Supplement

11. Main results

a) Report the associations between genetic variant and exposure, and between genetic variant and outcome, preferably on an interpretable scale (e.g. comparing 25th and 75th percentile of allele count or genetic risk score, if individual-level data available).

b) Report causal effect estimate between exposure and outcome, and the measures of uncertainty from the MR analysis. Use an intuitive scale, such as odds ratio, or relative risk, per standard deviation difference.

c) If relevant, consider translating estimates of relative risk into absolute risk for a meaningful time-period.

d) Consider any plots to visualize results (e.g. forest plot, scatterplot of associations between genetic variants and outcome versus between genetic variants and exposure).

Results

12. Assessment of assumptions

a) Assess the validity of the assumptions.

b) Report any additional statistics (e.g., assessments of heterogeneity, such as I2, Q statistic).

Results and Discussion

13. Sensitivity and additional analyses

a) Use sensitivity analyses to assess the robustness of the main results to violations of the assumptions.

b) Report results from other sensitivity analyses (e.g., replication study with different dataset, analyses of subgroups, validation of instrument(s), simulations, etc.).

c) Report any assessment of direction of causality (e.g., bidirectional MR).

d) When relevant, report and compare with estimates from non-MR analyses.

e) Consider any additional plots to visualize results (e.g., leave-one-out analyses).

Results and Discussion

DISCUSSION

14. Key results

Summarize key results with reference to study objectives.

Discussion

15. Limitations

Discuss limitations of the study, taking into account the validity of the MR assumptions, other sources of potential bias, and imprecision. Discuss both direction and magnitude of any potential bias, and any efforts to address them.

Discussion

16. Interpretation

a) Give a cautious overall interpretation of results considering objectives and limitations.

Compare with results from other relevant studies.

b) Discuss underlying biological mechanisms that could be modelled by using the genetic variants to assess the relationship between the exposure and the outcome.

c) Discuss whether the results have clinical or policy relevance, and whether interventions could have the same size effect.

Discussion

17. Generalizability

Discuss the generalizability of the study results (a) to other populations (i.e. external validity),

(b) across other exposure periods/timings, and (c) across other levels of exposure.

Discussion

OTHER INFORMATION

18. Funding

Give the source of funding and the role of the funders for the present study and, if applicable, for the original study or studies on which the present article is based.

Funding

19. Data and data sharing

Present data used to perform all analyses or report where and how the data can be accessed. State whether statistical code is publicly accessible and if so, where.

Methods

20. Conflicts of Interest

All authors should declare all potential conflicts of interest.

Conflicts of interest

Table S1. Baseline characteristics for the UK Biobank participants included in the genome-wide association study analyses of mean arterial pressure (MAP) ≤55 years, MAP >55years, and coronary artery disease (CAD) >55 years.

Variable	MAP ≤55 N=163,147	MAP >55 N=245,749	CAD > 55 Cases (N=8,788)	CAD > 55 Controls (N=184,201)
Age, mean (SD), y	48.5 (4.4)	62.5 (3.8)	62.2 (5.0)	58.2 (6.6)
Sex, N (%)				
Male	72,657 (44.5)	115,229 (46.9)	5,791 (65.9)	79,391 (43.1)
Female	90,490 (55.5)	130,520 (53.1)	2,997 (34.1)	104,810 (56.9)
Systolic blood pressure, mmHg (SD)	132.0 (16.7)	142.6 (18.7)	145.9 (19.1)	139.6 (18.6)
Diastolic blood pressure, mmHg (SD)	81.9 (10.3)	82.6 (9.9)	83.8 (10.6)	82.8 (10.0)
BMI,kg/m^2 (SD)	27.2 (5.0)	27.6 (4.6)	28.9 (4.9)	27.4 (4.7)
Current smokers, N (%)	20,440 (12.5)	20,880 (8.5)	1,024 (11.6)	14,486 (7.9)
Antihypertensive medication, N (%)	15,336 (9.4)	70,038 (28.5)	3,612 (41.1)	36,840 (20.0)

Table S2. Instruments and genetic association estimates for Model 1 of the univariable Mendelian randomization analysis. GX: genetic association estimate with MAP ≤55 years (mmHg); GY: genetic association estimate with coronary artery disease (log odds ratio); SE: standard error; SNP: single-nucleotide polymorphism.

SNP	Effect Allele	GX	GX_SE	GX_P	GY	GY_SE	GY_P
rs1057040	G	0.256	0.043	2.36E-09	0.008	0.009	3.90E-01
rs10993958	A	-0.652	0.081	6.02E-16	-0.041	0.028	1.42E-01
rs10995311	G	-0.290	0.043	2.39E-11	0.011	0.010	2.37E-01
rs11039144	A	-0.385	0.059	7.93E-11	0.003	0.012	7.75E-01
rs11070245	T	-0.237	0.043	3.73E-08	0.005	0.009	5.89E-01
rs11072508	C	0.517	0.046	2.94E-29	0.020	0.010	4.99E-02
rs11187838	A	-0.367	0.043	2.28E-17	0.024	0.009	9.21E-03
rs113397083	A	-0.616	0.084	1.68E-13	-0.048	0.017	4.30E-03
rs115262049	T	-0.433	0.076	1.28E-08	-0.032	0.020	1.08E-01
rs11642015	T	0.324	0.044	1.39E-13	0.030	0.010	1.78E-03
rs11669915	G	0.334	0.049	1.16E-11	0.038	0.011	5.53E-04
rs11721038	C	-0.487	0.082	2.95E-09	-0.046	0.015	2.53E-03
rs12137438	C	0.251	0.044	1.02E-08	0.008	0.009	3.95E-01
rs1216743	G	-0.605	0.048	2.31E-36	-0.031	0.010	2.34E-03
rs12185567	A	-0.276	0.048	7.88E-09	0.005	0.011	6.47E-01
rs12258967	G	-0.539	0.047	2.89E-30	-0.026	0.011	2.15E-02
rs12363520	A	0.293	0.052	1.75E-08	-0.023	0.013	8.61E-02
rs12627514	G	0.288	0.048	1.42E-09	0.007	0.011	5.39E-01
rs12644723	G	0.262	0.044	3.01E-09	0.021	0.009	2.49E-02
rs12656497	T	-0.504	0.044	7.43E-31	-0.011	0.009	2.39E-01
rs12693302	G	0.324	0.045	5.22E-13	0.035	0.010	3.73E-04
rs12716338	A	-0.363	0.045	7.15E-16	-0.024	0.010	1.47E-02
rs1275988	C	0.387	0.044	1.82E-18	0.003	0.009	7.50E-01
rs13112725	G	-0.385	0.050	1.69E-14	-0.001	0.011	9.48E-01
rs13121442	T	-0.280	0.043	7.16E-11	-0.037	0.009	9.18E-05
rs13125101	A	0.831	0.047	3.36E-69	0.048	0.010	3.02E-06
rs13163533	G	0.447	0.080	1.92E-08	0.015	0.019	4.26E-01
rs14235	A	-0.244	0.045	4.77E-08	-0.008	0.010	4.13E-01
rs145339349	A	1.225	0.162	4.19E-14	0.036	0.043	4.08E-01
rs167479	T	-0.533	0.043	2.61E-35	-0.015	0.014	2.79E-01
rs17173238	G	0.269	0.048	1.58E-08	0.015	0.011	1.72E-01
rs17257695	G	-0.325	0.059	4.60E-08	-0.010	0.014	4.60E-01
rs17637472	A	0.285	0.044	1.08E-10	0.041	0.010	5.64E-05
rs17677603	G	0.250	0.044	1.56E-08	0.028	0.010	3.25E-03
rs17732246	A	0.384	0.064	2.37E-09	0.019	0.014	1.67E-01
rs1801253	G	-0.442	0.049	1.82E-19	-0.015	0.011	1.70E-01
rs1887320	A	0.362	0.043	4.20E-17	0.023	0.009	1.30E-02
rs1896326	A	-0.283	0.052	5.00E-08	-0.004	0.012	7.23E-01
rs1966697	C	0.320	0.053	1.49E-09	0.008	0.011	4.91E-01
rs1980235	G	-0.447	0.046	1.64E-22	0.032	0.010	1.49E-03
rs200538	C	-0.320	0.048	3.66E-11	-0.003	0.010	7.92E-01
rs2032451	T	0.523	0.060	1.89E-18	0.008	0.014	5.95E-01
rs2163379	A	0.369	0.045	1.33E-16	0.008	0.010	4.00E-01
rs2246832	A	-0.291	0.043	1.11E-11	-0.030	0.009	1.74E-03
rs2294239	G	-0.240	0.043	3.45E-08	-0.019	0.009	4.62E-02
rs2301597	T	0.352	0.044	5.78E-16	0.000	0.009	9.82E-01
rs2306363	T	-0.344	0.053	9.90E-11	-0.050	0.012	1.96E-05
rs2443708	T	-0.283	0.046	9.15E-10	-0.013	0.010	1.97E-01
rs2478531	C	0.329	0.044	5.54E-14	0.018	0.025	4.72E-01
rs2644128	C	-0.244	0.043	1.51E-08	-0.021	0.009	2.29E-02
rs2645158	A	0.251	0.044	1.24E-08	0.016	0.010	8.53E-02
rs268263	T	-0.526	0.050	1.15E-25	-0.042	0.011	1.16E-04
rs2724486	C	-0.272	0.049	2.31E-08	-0.029	0.011	6.93E-03
rs28416181	G	-0.356	0.050	6.62E-13	-0.020	0.011	6.31E-02
rs28866311	G	0.334	0.043	7.01E-15	0.014	0.009	1.34E-01

rs2947411	A	-0.349	0.057	7.20E-10	-0.035	0.012	4.09E-03
rs2969072	A	0.329	0.046	6.77E-13	-0.006	0.011	6.10E-01
rs2978098	C	-0.237	0.043	4.02E-08	-0.004	0.009	7.01E-01
rs3096009	G	0.261	0.046	1.16E-08	0.011	0.010	2.85E-01
rs3118905	A	0.284	0.048	2.71E-09	0.014	0.011	2.09E-01
rs34394882	T	0.276	0.047	4.32E-09	-0.006	0.011	5.70E-01
rs35213536	T	0.328	0.050	5.83E-11	0.019	0.012	1.03E-01
rs35429	G	-0.281	0.044	2.01E-10	0.002	0.010	8.74E-01
rs360153	T	-0.328	0.043	4.73E-14	-0.051	0.009	5.16E-08
rs3790604	A	0.490	0.082	2.60E-09	0.014	0.016	3.93E-01
rs389883	G	-0.344	0.046	5.20E-14	-0.027	0.012	2.22E-02
rs3936510	T	0.328	0.053	8.78E-10	0.042	0.012	4.73E-04
rs4076877	T	-0.590	0.097	1.33E-09	-0.067	0.033	4.41E-02
rs4077158	T	-0.265	0.043	7.37E-10	-0.012	0.009	1.96E-01
rs4147111	C	-0.498	0.084	3.09E-09	-0.067	0.019	3.61E-04
rs419076	T	0.341	0.043	2.17E-15	0.017	0.009	6.69E-02
rs4362428	A	-0.269	0.044	6.26E-10	-0.003	0.010	7.59E-01
rs4428270	C	0.259	0.044	3.42E-09	0.022	0.010	2.34E-02
rs45475403	T	-0.536	0.094	1.09E-08	-0.053	0.023	1.83E-02
rs4588930	A	-0.244	0.043	1.91E-08	-0.006	0.009	5.37E-01
rs4712656	C	0.270	0.043	4.23E-10	0.008	0.009	3.95E-01
rs4766578	T	0.522	0.043	6.07E-34	0.066	0.011	2.83E-10
rs4980379	T	0.310	0.045	4.14E-12	0.013	0.010	2.00E-01
rs5068	G	-1.091	0.094	3.70E-31	-0.005	0.022	8.30E-01
rs56179563	A	-0.303	0.044	8.97E-12	-0.067	0.011	7.46E-10
rs56256623	A	-0.357	0.055	1.05E-10	-0.063	0.012	1.32E-07
rs56313611	T	-0.388	0.062	3.27E-10	-0.061	0.014	1.10E-05
rs568546	C	0.261	0.043	1.48E-09	0.019	0.009	3.85E-02
rs57786342	A	0.335	0.053	3.56E-10	0.015	0.012	2.10E-01
rs6026739	T	0.592	0.067	9.28E-19	0.052	0.015	3.67E-04
rs6039212	C	-0.290	0.051	1.51E-08	-0.003	0.011	7.57E-01
rs62036942	C	-0.388	0.062	4.32E-10	0.003	0.013	7.96E-01
rs62052380	T	0.363	0.066	3.18E-08	0.012	0.020	5.47E-01
rs62104477	T	0.274	0.046	2.09E-09	-0.023	0.010	2.51E-02
rs62445396	T	0.715	0.127	1.96E-08	0.018	0.031	5.53E-01
rs6438857	C	-0.251	0.044	8.15E-09	-0.011	0.009	2.47E-01
rs6798940	A	-0.248	0.043	8.44E-09	0.002	0.009	8.46E-01
rs6923947	A	0.347	0.043	1.05E-15	0.007	0.009	4.58E-01
rs6990531	G	-0.352	0.050	2.62E-12	-0.001	0.010	9.06E-01
rs7067916	A	0.288	0.050	8.13E-09	0.017	0.011	1.07E-01
rs7070797	A	-0.562	0.062	7.76E-20	-0.013	0.015	4.03E-01
rs7121365	A	0.444	0.065	8.81E-12	0.025	0.014	8.53E-02
rs7125196	C	-0.447	0.067	3.15E-11	-0.017	0.013	1.77E-01
rs71508634	T	-0.256	0.047	3.96E-08	0.005	0.010	6.40E-01
rs71654213	T	-0.299	0.044	1.57E-11	0.023	0.011	3.75E-02
rs7171632	C	0.246	0.043	1.30E-08	0.012	0.010	2.35E-01
rs72654647	A	0.328	0.050	5.70E-11	0.026	0.011	1.56E-02
rs72677850	A	-1.216	0.162	5.08E-14	-0.072	0.040	7.31E-02
rs72812818	C	-0.276	0.047	3.37E-09	-0.011	0.011	3.13E-01
rs72854462	G	0.288	0.050	7.20E-09	-0.025	0.011	2.55E-02
rs7302981	A	0.374	0.044	3.21E-17	0.000	0.010	9.76E-01
rs73046792	A	-0.352	0.058	1.13E-09	-0.016	0.016	3.17E-01
rs73075659	G	-0.411	0.045	1.10E-19	-0.006	0.011	5.56E-01
rs73234219	T	0.244	0.044	2.40E-08	-0.013	0.010	1.68E-01
rs741066	T	0.266	0.047	1.50E-08	0.036	0.011	7.22E-04
rs74233809	C	-0.771	0.080	4.48E-22	-0.072	0.014	4.09E-07
rs74661587	G	0.525	0.063	1.07E-16	0.017	0.013	1.75E-01
rs748676	A	0.543	0.082	3.26E-11	0.007	0.013	5.90E-01
rs75034121	C	-0.413	0.074	2.33E-08	0.007	0.017	6.53E-01
rs7604588	A	0.265	0.047	2.30E-08	0.024	0.010	2.26E-02
rs7753358	A	0.292	0.044	2.16E-11	0.028	0.009	3.57E-03
rs7845053	A	-0.256	0.044	5.44E-09	0.010	0.010	3.12E-01
rs79780963	T	-0.766	0.080	1.51E-21	-0.077	0.014	7.54E-08

rs79997166	C	0.537	0.090	2.62E-09	0.011	0.025	6.64E-01
rs8027450	T	0.487	0.046	4.74E-26	0.057	0.011	1.35E-07
rs8070460	C	-0.268	0.043	4.89E-10	-0.040	0.009	2.06E-05
rs891511	A	-0.437	0.047	5.35E-21	-0.021	0.011	4.92E-02
rs9667542	A	-0.310	0.054	8.13E-09	0.001	0.014	9.58E-01
rs9719973	A	0.327	0.044	8.66E-14	0.011	0.010	2.44E-01

Table S3. Instruments and genetic association estimates for Model 2 of the univariable Mendelian randomization analysis. GX: genetic association estimate with MAP ≤55 years (mmHg); GY: genetic association estimate with coronary artery disease (log odds ratio); SE: standard error; SNP: single-nucleotide polymorphism.

SNP	Effect Allele	GX	GX_SE	GX_P	GY	GY_SE	GY_P
rs1057040	G	-0.455	0.115	7.76E-05	-0.016	0.034	6.45E-01
rs10993958	A	-0.493	0.084	5.30E-09	-0.016	0.025	5.17E-01
rs10995311	G	-0.296	0.059	5.86E-07	0.016	0.017	3.62E-01
rs11039144	A	-0.425	0.059	6.66E-13	0.024	0.017	1.61E-01
rs11070245	T	-0.824	0.109	4.53E-14	-0.001	0.032	9.74E-01
rs11072508	C	-0.819	0.110	8.92E-14	0.001	0.032	9.87E-01
rs11187838	A	-0.531	0.067	2.44E-15	-0.038	0.020	5.27E-02
rs113397083	A	0.271	0.068	7.42E-05	0.027	0.020	1.77E-01
rs115262049	T	-0.523	0.064	4.62E-16	-0.014	0.019	4.58E-01
rs11642015	T	0.285	0.061	3.19E-06	0.033	0.018	6.54E-02
rs11669915	G	-0.394	0.092	1.94E-05	-0.034	0.027	2.12E-01
rs11721038	C	-0.322	0.072	9.05E-06	-0.024	0.021	2.69E-01
rs12137438	C	-0.600	0.066	8.17E-20	-0.050	0.019	9.94E-03
rs1216743	G	0.247	0.059	2.87E-05	0.035	0.017	4.30E-02
rs12185567	A	0.374	0.089	2.54E-05	0.075	0.026	3.34E-03
rs12258967	G	-0.304	0.060	3.41E-07	-0.050	0.017	4.18E-03
rs12363520	A	0.256	0.071	3.06E-04	-0.011	0.021	5.94E-01
rs12627514	G	0.322	0.072	8.84E-06	0.009	0.021	6.88E-01
rs12644723	G	-0.350	0.081	1.52E-05	0.015	0.024	5.21E-01
rs12656497	T	-0.420	0.073	1.12E-08	-0.050	0.022	2.07E-02
rs12693302	G	-0.497	0.063	2.03E-15	0.006	0.018	7.30E-01
rs12716338	A	0.488	0.059	1.21E-16	0.085	0.017	6.31E-07
rs1275988	C	-0.318	0.071	7.76E-06	-0.038	0.021	6.76E-02
rs13112725	G	-0.260	0.060	1.73E-05	0.019	0.018	2.69E-01
rs13121442	T	-0.411	0.062	3.26E-11	-0.017	0.018	3.60E-01
rs13125101	A	0.417	0.061	5.55E-12	-0.012	0.018	4.85E-01
rs13163533	G	0.280	0.065	1.87E-05	0.026	0.019	1.77E-01
rs14235	A	0.539	0.112	1.44E-06	0.019	0.033	5.57E-01
rs145339349	A	-1.000	0.222	6.50E-06	-0.063	0.066	3.44E-01
rs167479	T	0.251	0.073	5.84E-04	0.004	0.021	8.69E-01
rs17173238	G	0.482	0.063	2.44E-14	0.074	0.018	5.33E-05
rs17257695	G	0.416	0.088	2.41E-06	0.004	0.026	8.72E-01
rs17637472	A	-0.212	0.059	3.26E-04	-0.036	0.017	3.88E-02
rs17677603	G	0.333	0.059	1.44E-08	0.017	0.017	3.13E-01
rs17732246	A	0.523	0.063	1.00E-16	0.019	0.018	2.98E-01
rs1801253	G	0.222	0.059	1.79E-04	0.010	0.017	5.77E-01
rs1887320	A	-0.471	0.085	2.62E-08	-0.022	0.025	3.80E-01
rs1896326	A	-0.335	0.066	4.20E-07	0.005	0.019	7.90E-01
rs1966697	C	-0.307	0.061	5.30E-07	0.009	0.018	6.12E-01
rs1980235	G	0.311	0.060	2.15E-07	0.020	0.017	2.63E-01
rs200538	C	0.336	0.090	1.81E-04	-0.036	0.027	1.73E-01
rs2032451	T	-0.246	0.060	3.78E-05	0.000	0.017	9.85E-01
rs2163379	A	0.262	0.059	8.18E-06	0.028	0.017	1.07E-01
rs2246832	A	0.391	0.060	5.19E-11	0.007	0.017	7.00E-01
rs2294239	G	0.236	0.060	8.73E-05	0.033	0.018	6.05E-02
rs2301597	T	-0.287	0.059	1.16E-06	-0.016	0.017	3.42E-01
rs2306363	T	0.376	0.064	5.69E-09	-0.003	0.019	8.68E-01
rs2443708	T	-0.547	0.059	1.18E-20	-0.051	0.017	2.83E-03
rs2478531	C	0.245	0.063	9.14E-05	0.025	0.018	1.78E-01
rs2644128	C	0.265	0.060	1.09E-05	-0.020	0.018	2.52E-01
rs2645158	A	0.329	0.067	1.01E-06	0.053	0.019	6.90E-03
rs268263	T	-0.383	0.079	1.25E-06	0.000	0.023	9.95E-01
rs2724486	C	0.538	0.112	1.70E-06	0.004	0.033	8.99E-01
rs28416181	G	1.433	0.217	4.05E-11	0.123	0.063	5.06E-02
rs28866311	G	-0.331	0.129	1.00E-02	0.024	0.037	5.08E-01

rs2947411	A	-1.193	0.129	2.93E-20	-0.035	0.038	3.65E-01
rs2969072	A	0.234	0.060	9.21E-05	0.017	0.017	3.40E-01
rs2978098	C	-0.293	0.059	6.54E-07	-0.001	0.017	9.61E-01
rs3096009	G	0.358	0.060	2.31E-09	0.027	0.017	1.24E-01
rs3118905	A	0.321	0.068	2.69E-06	-0.009	0.020	6.53E-01
rs34394882	T	-0.318	0.061	1.67E-07	0.018	0.018	3.03E-01
rs35213536	T	-0.293	0.070	3.09E-05	-0.021	0.021	2.95E-01
rs35429	G	0.374	0.059	2.23E-10	0.007	0.017	6.65E-01
rs360153	T	-0.439	0.085	2.16E-07	-0.047	0.025	6.20E-02
rs3790604	A	0.728	0.091	1.63E-15	0.060	0.026	2.25E-02
rs389883	G	0.345	0.069	4.73E-07	0.012	0.020	5.50E-01
rs3936510	T	0.362	0.065	2.80E-08	0.020	0.019	2.84E-01
rs4076877	T	-0.282	0.059	2.07E-06	-0.017	0.017	3.19E-01
rs4077158	T	-0.400	0.077	2.44E-07	0.031	0.022	1.70E-01
rs4147111	C	0.262	0.065	5.19E-05	-0.024	0.019	2.12E-01
rs419076	T	0.362	0.068	1.02E-07	-0.023	0.020	2.45E-01
rs4362428	A	0.429	0.124	5.28E-04	0.020	0.036	5.66E-01
rs4428270	C	-0.454	0.069	3.86E-11	-0.028	0.020	1.70E-01
rs45475403	T	-0.300	0.065	4.52E-06	0.022	0.019	2.49E-01
rs4588930	A	0.328	0.061	9.75E-08	0.019	0.018	2.82E-01
rs4712656	C	-0.444	0.081	4.77E-08	-0.007	0.024	7.63E-01
rs4766578	T	-0.327	0.101	1.27E-03	-0.022	0.030	4.67E-01
rs4980379	T	0.455	0.060	5.18E-14	0.016	0.018	3.58E-01
rs5068	G	-0.405	0.105	1.13E-04	0.024	0.030	4.28E-01
rs56179563	A	-0.286	0.059	1.58E-06	-0.020	0.018	2.53E-01
rs56256623	A	-0.280	0.059	2.03E-06	-0.015	0.017	3.81E-01
rs56313611	T	-0.354	0.059	1.93E-09	-0.012	0.017	4.93E-01
rs568546	C	-0.420	0.112	1.85E-04	-0.001	0.033	9.81E-01
rs57786342	A	0.380	0.059	1.07E-10	0.012	0.017	4.94E-01
rs6026739	T	-0.335	0.063	1.15E-07	-0.055	0.019	3.18E-03
rs6039212	C	-0.282	0.059	1.58E-06	-0.024	0.017	1.56E-01
rs62036942	C	0.799	0.065	7.11E-35	0.063	0.019	8.00E-04
rs62052380	T	0.279	0.060	4.08E-06	0.021	0.018	2.31E-01
rs62104477	T	-0.398	0.069	6.41E-09	-0.017	0.020	4.03E-01
rs62445396	T	-0.321	0.059	4.88E-08	-0.025	0.017	1.42E-01
rs6438857	C	-0.385	0.076	3.60E-07	-0.071	0.022	1.71E-03
rs6798940	A	-0.351	0.067	1.45E-07	-0.020	0.020	2.98E-01
rs6923947	A	0.205	0.060	6.25E-04	0.010	0.017	5.53E-01
rs6990531	G	0.296	0.060	7.60E-07	0.027	0.017	1.14E-01
rs7067916	A	0.557	0.109	3.22E-07	0.008	0.032	7.92E-01
rs7070797	A	0.470	0.087	5.49E-08	0.013	0.025	6.00E-01
rs7121365	A	0.235	0.061	1.03E-04	0.016	0.018	3.69E-01
rs7125196	C	0.235	0.063	1.72E-04	0.010	0.018	5.90E-01
rs71508634	T	-0.416	0.061	1.25E-11	-0.011	0.018	5.38E-01
rs71654213	T	-0.208	0.064	1.13E-03	-0.039	0.019	3.79E-02
rs7171632	C	-0.474	0.060	2.08E-15	0.000	0.017	9.77E-01
rs72654647	A	0.339	0.073	3.66E-06	0.022	0.021	3.09E-01
rs72677850	A	0.351	0.060	4.31E-09	0.012	0.017	4.91E-01
rs72812818	C	0.399	0.059	1.63E-11	-0.026	0.017	1.37E-01
rs72854462	G	-0.719	0.115	3.86E-10	-0.049	0.034	1.44E-01
rs7302981	A	0.207	0.059	4.49E-04	-0.001	0.017	9.31E-01
rs73046792	A	0.475	0.082	6.53E-09	-0.018	0.024	4.41E-01
rs73075659	G	-0.339	0.063	5.83E-08	-0.023	0.018	2.15E-01
rs73234219	T	0.302	0.063	1.52E-06	0.019	0.018	3.04E-01
rs741066	T	-0.310	0.061	3.50E-07	-0.030	0.018	8.84E-02
rs74233809	C	0.270	0.060	6.59E-06	0.034	0.017	5.16E-02
rs74661587	G	0.259	0.064	5.71E-05	0.028	0.019	1.27E-01
rs748676	A	-0.516	0.064	6.00E-16	-0.004	0.019	8.41E-01
rs75034121	C	0.267	0.065	3.82E-05	0.008	0.019	6.86E-01
rs7604588	A	0.913	0.174	1.60E-07	0.053	0.050	2.95E-01
rs7753358	A	-0.275	0.069	6.58E-05	-0.022	0.020	2.73E-01
rs7845053	A	-0.259	0.059	1.16E-05	0.013	0.017	4.46E-01
rs79780963	T	-0.266	0.060	9.65E-06	-0.025	0.018	1.57E-01

rs79997166	C	-0.588	0.133	1.02E-05	0.012	0.039	7.65E-01
rs8027450	T	0.385	0.061	3.08E-10	0.037	0.018	3.90E-02
rs8070460	C	-0.303	0.068	7.78E-06	-0.014	0.020	4.95E-01
rs891511	A	-0.284	0.059	1.64E-06	0.005	0.017	7.64E-01
rs9667542	A	-0.624	0.111	1.80E-08	-0.071	0.033	3.19E-02
rs9719973	A	-0.280	0.064	1.12E-05	-0.011	0.019	5.62E-01

Table S4. Instruments and genetic association estimates for Models 1 and 3 of the multivariable Mendelian randomization analysis. GX: genetic association estimate with MAP ≤55 years (mmHg); GY: genetic association estimate with coronary artery disease (log odds ratio); GZ: genetic association estimates with MAP >55 years (mmHg); SE: standard error; SNP: single-nucleotide polymorphism.

SNP	Effect Allele	GX	GX_SE	GX_P	GZ	GZ_SE	GZ_P	GY	GY_SE	GY_P	GX and GZ mean	GX and GZ difference	Included in Model 3
rs1000423	C	-0.15	0.05	2.1E-03	-0.33	0.04	9.0E-15	-0.04	0.01	1.4E-04	-0.24	0.18	No
rs1010064	C	-0.22	0.06	9.5E-05	-0.27	0.05	1.9E-08	-0.04	0.01	4.1E-04	-0.24	0.05	No
rs10119435	A	0.42	0.13	8.1E-04	0.62	0.11	1.7E-08	0.03	0.03	2.3E-01	0.52	-0.20	Yes
rs10158537	G	0.23	0.05	9.5E-07	0.30	0.04	7.9E-14	0.00	0.01	7.6E-01	0.27	-0.07	No
rs10226118	C	-0.06	0.04	1.7E-01	-0.25	0.04	6.8E-11	0.00	0.01	7.0E-01	-0.15	0.19	No
rs10248237	A	0.26	0.10	5.9E-03	0.46	0.08	2.7E-08	0.03	0.02	1.8E-01	0.36	-0.20	Yes
rs10265221	C	0.28	0.05	4.3E-09	0.29	0.04	1.0E-12	0.01	0.01	3.8E-01	0.29	-0.02	No
rs10409243	C	0.11	0.04	1.3E-02	0.21	0.04	3.7E-08	0.01	0.01	1.6E-01	0.16	-0.10	No
rs10468291	C	0.09	0.04	4.0E-02	0.22	0.04	6.5E-09	0.00	0.01	6.1E-01	0.15	-0.13	No
rs10777213	G	0.26	0.04	7.1E-10	0.15	0.04	5.6E-05	0.00	0.01	7.0E-01	0.21	0.11	No
rs10852034	T	-0.16	0.04	2.2E-04	-0.23	0.04	1.2E-09	0.00	0.01	6.9E-01	-0.20	0.07	No
rs10858071	A	1.09	0.15	2.7E-13	0.65	0.13	8.2E-07	0.01	0.04	8.1E-01	0.87	0.45	Yes
rs10876531	C	-0.21	0.05	1.2E-05	-0.27	0.04	4.9E-11	-0.02	0.01	6.5E-02	-0.24	0.07	No
rs10900127	C	0.08	0.04	7.8E-02	0.22	0.04	6.5E-09	-0.01	0.01	1.9E-01	0.15	-0.14	No
rs10993958	A	-0.65	0.08	6.0E-16	-0.42	0.07	2.7E-09	-0.04	0.03	1.4E-01	-0.54	-0.23	Yes
rs10995307	T	0.28	0.04	4.7E-11	-0.31	0.04	1.1E-16	0.00	0.01	8.6E-01	-0.01	0.59	Yes
rs11070245	T	-0.24	0.04	3.7E-08	-0.20	0.04	1.0E-07	0.01	0.01	5.9E-01	-0.22	-0.04	No
rs11072508	C	0.52	0.05	2.9E-29	0.32	0.04	9.8E-16	0.02	0.01	5.0E-02	0.42	0.20	Yes
rs1114348	A	0.22	0.04	4.2E-07	0.25	0.04	1.3E-11	0.03	0.01	5.5E-03	0.23	-0.04	No
rs11187838	A	-0.37	0.04	2.3E-17	-0.40	0.04	6.1E-26	0.02	0.01	9.2E-03	-0.38	0.03	No
rs113044050	T	-0.24	0.06	1.3E-04	-0.37	0.05	8.1E-12	-0.02	0.01	7.4E-02	-0.30	0.13	No
rs113230003	A	-0.29	0.05	2.6E-09	-0.19	0.04	1.1E-05	-0.02	0.01	1.5E-01	-0.24	-0.10	No
rs1133400	G	0.17	0.05	9.3E-04	0.29	0.05	1.3E-10	0.01	0.01	3.7E-01	0.23	-0.12	No
rs113458760	G	-0.15	0.04	1.2E-03	-0.24	0.04	1.5E-09	-0.02	0.01	2.3E-01	-0.19	0.09	No
rs113695818	T	-0.14	0.05	2.5E-03	-0.29	0.04	1.2E-12	-0.01	0.01	5.3E-01	-0.21	0.15	No
rs115262049	T	-0.43	0.08	1.3E-08	-0.48	0.07	3.2E-13	-0.03	0.02	1.1E-01	-0.46	0.05	No
rs11642015	T	0.32	0.04	1.4E-13	0.09	0.04	1.9E-02	0.03	0.01	1.8E-03	0.21	0.23	Yes
rs11669915	G	0.33	0.05	1.2E-11	0.23	0.04	1.4E-07	0.04	0.01	5.5E-04	0.28	0.11	No
rs11676040	C	0.09	0.05	6.6E-02	0.25	0.04	7.9E-10	0.02	0.01	3.8E-02	0.17	-0.17	No
rs11749673	G	-0.13	0.05	7.0E-03	-0.25	0.04	5.7E-09	-0.02	0.01	4.3E-02	-0.19	0.12	No
rs1175651	T	0.24	0.05	3.8E-06	0.26	0.05	2.6E-08	0.00	0.01	7.9E-01	0.25	-0.01	No
rs11760498	A	0.23	0.06	7.2E-05	0.28	0.05	1.1E-08	0.01	0.01	4.0E-01	0.26	-0.06	No
rs11774829	A	-0.28	0.07	8.0E-05	-0.38	0.06	9.4E-10	-0.02	0.02	2.3E-01	-0.33	0.10	No
rs11821781	G	0.25	0.05	5.8E-07	0.32	0.04	1.4E-13	0.00	0.01	7.2E-01	0.28	-0.07	No
rs11915142	A	0.20	0.04	3.7E-06	0.25	0.04	9.3E-11	0.01	0.01	1.4E-01	0.23	-0.04	No
rs12057453	T	0.22	0.04	7.6E-07	0.24	0.04	2.4E-10	0.01	0.01	1.7E-01	0.23	-0.03	No
rs12137438	C	0.25	0.04	1.0E-08	0.10	0.04	9.2E-03	0.01	0.01	4.0E-01	0.18	0.15	No
rs12194642	A	0.25	0.04	1.5E-08	0.26	0.04	2.0E-11	0.02	0.01	2.3E-02	0.25	-0.01	No
rs12216886	G	-0.08	0.06	1.3E-01	-0.27	0.05	1.5E-08	0.00	0.01	7.1E-01	-0.18	0.19	No
rs12258967	G	-0.54	0.05	2.9E-30	-0.39	0.04	7.4E-22	-0.03	0.01	2.2E-02	-0.47	-0.15	No
rs1229984	T	-0.83	0.15	1.7E-08	-0.61	0.12	1.2E-06	-0.03	0.02	7.5E-02	-0.72	-0.22	Yes
rs12567136	T	-0.67	0.06	8.9E-31	-0.65	0.05	2.4E-38	-0.02	0.01	7.0E-02	-0.66	-0.01	No
rs12627514	G	0.29	0.05	1.4E-09	0.17	0.04	5.8E-05	0.01	0.01	5.4E-01	0.23	0.12	No
rs12643599	G	-0.27	0.04	2.9E-09	-0.27	0.04	3.1E-12	-0.05	0.01	7.1E-08	-0.27	0.01	No
rs12656497	T	-0.50	0.04	7.4E-31	-0.47	0.04	4.5E-35	-0.01	0.01	2.4E-01	-0.49	-0.03	No
rs12693302	G	0.32	0.04	5.2E-13	0.22	0.04	1.0E-08	0.03	0.01	3.7E-04	0.27	0.10	No
rs1275978	C	0.38	0.04	4.6E-18	0.48	0.04	4.4E-35	0.00	0.01	9.1E-01	0.43	-0.09	No
rs12978472	G	-0.56	0.06	8.1E-18	-0.72	0.06	8.4E-38	-0.02	0.02	2.2E-01	-0.64	0.16	No
rs12983032	A	-0.13	0.05	2.9E-03	-0.23	0.04	8.1E-09	-0.03	0.01	3.3E-03	-0.18	0.09	No
rs13107325	T	-0.48	0.08	5.7E-09	-0.64	0.07	2.7E-19	-0.01	0.02	7.7E-01	-0.56	0.16	No
rs13121442	T	-0.28	0.04	7.2E-11	-0.20	0.04	6.5E-08	-0.04	0.01	9.2E-05	-0.24	-0.08	No
rs13125101	A	0.83	0.05	3.4E-69	0.57	0.04	2.0E-44	0.05	0.01	3.0E-06	0.70	0.26	Yes

rs13227860	A	0.14	0.05	3.8E-03	0.25	0.04	1.1E-09	0.02	0.01	1.0E-01	0.19	-0.11	No
rs13324341	T	0.14	0.06	1.9E-02	0.28	0.05	4.5E-08	0.07	0.01	4.3E-09	0.21	-0.14	No
rs1436049	G	-0.13	0.05	7.1E-03	-0.24	0.04	1.4E-08	-0.01	0.01	3.6E-01	-0.19	0.11	No
rs1436138	G	-0.18	0.04	3.8E-05	-0.28	0.04	1.0E-12	-0.01	0.01	4.1E-01	-0.23	0.10	No
rs1472467	G	-0.14	0.04	1.2E-03	-0.21	0.04	3.4E-08	0.02	0.01	1.3E-02	-0.17	0.07	No
rs1487629	A	0.17	0.04	5.1E-05	0.25	0.04	2.9E-11	0.00	0.01	6.2E-01	0.21	-0.08	No
rs1492027	G	0.14	0.05	8.7E-03	0.29	0.05	2.0E-10	0.00	0.01	7.7E-01	0.21	-0.15	No
rs150857355	C	0.54	0.15	2.6E-04	0.80	0.13	8.3E-10	0.08	0.04	7.8E-02	0.67	-0.26	Yes
rs1544861	T	0.32	0.05	3.9E-12	0.16	0.04	4.0E-05	-0.01	0.01	2.7E-01	0.24	0.15	No
rs164101	G	-0.27	0.05	9.5E-09	-0.19	0.04	5.7E-06	-0.01	0.01	4.0E-01	-0.23	-0.08	No
rs1687318	T	0.25	0.05	2.6E-07	0.28	0.04	1.7E-11	0.01	0.01	2.6E-01	0.27	-0.03	No
rs16895971	C	0.30	0.06	1.7E-06	0.35	0.05	1.6E-10	0.02	0.01	1.0E-01	0.32	-0.05	No
rs17249754	A	-0.48	0.06	1.8E-17	-0.52	0.05	9.2E-26	0.07	0.01	2.1E-10	-0.50	0.04	No
rs17257695	G	-0.32	0.06	4.6E-08	-0.11	0.05	3.8E-02	-0.01	0.01	4.6E-01	-0.22	-0.22	Yes
rs17517959	C	0.13	0.04	4.8E-03	0.22	0.04	8.9E-09	0.00	0.01	8.7E-01	0.17	-0.10	No
rs17637472	A	0.28	0.04	1.1E-10	0.23	0.04	4.4E-09	0.04	0.01	5.6E-05	0.26	0.06	No
rs17717829	C	-0.18	0.04	4.5E-05	-0.27	0.04	3.0E-13	-0.01	0.01	4.9E-01	-0.23	0.10	No
rs17762	A	0.34	0.08	5.3E-05	0.41	0.07	2.4E-08	0.01	0.01	3.3E-01	0.38	-0.07	No
rs1801253	G	-0.44	0.05	1.8E-19	-0.35	0.04	5.5E-16	-0.01	0.01	1.7E-01	-0.39	-0.10	No
rs187680068	G	-0.21	0.04	1.4E-06	-0.24	0.04	3.6E-10	0.04	0.04	3.8E-01	-0.23	0.03	No
rs1896326	A	-0.28	0.05	5.0E-08	-0.17	0.05	1.8E-04	0.00	0.01	7.2E-01	-0.23	-0.11	No
rs1958603	A	-0.35	0.04	1.9E-15	-0.33	0.04	4.3E-17	-0.02	0.01	1.4E-02	-0.34	-0.03	No
rs2014590	T	-0.31	0.04	3.5E-13	-0.33	0.04	7.3E-19	-0.02	0.01	3.3E-02	-0.32	0.02	No
rs2032451	T	0.52	0.06	1.9E-18	0.44	0.05	3.8E-17	0.01	0.01	5.9E-01	0.48	0.08	No
rs2050905	G	0.11	0.04	1.3E-02	0.23	0.04	2.4E-09	0.00	0.01	9.8E-01	0.17	-0.12	No
rs2067832	G	0.08	0.04	6.4E-02	0.24	0.04	3.1E-10	-0.02	0.01	1.3E-02	0.16	-0.16	No
rs2071265	C	-0.37	0.08	5.8E-06	-0.79	0.07	3.3E-28	0.01	0.02	5.6E-01	-0.58	0.41	Yes
rs2073641	A	0.16	0.08	3.0E-02	0.38	0.07	7.6E-09	0.01	0.02	7.9E-01	0.27	-0.22	Yes
rs2087319	A	-0.12	0.05	1.8E-02	-0.27	0.04	1.2E-09	-0.02	0.01	3.1E-02	-0.19	0.15	No
rs2165301	C	0.15	0.04	7.0E-04	0.26	0.04	2.4E-11	0.02	0.01	1.5E-02	0.20	-0.11	No
rs2178270	C	-0.23	0.06	4.8E-05	-0.29	0.05	2.0E-09	-0.03	0.01	3.0E-02	-0.26	0.06	No
rs2242338	C	-0.48	0.08	9.4E-09	-0.38	0.07	1.6E-07	-0.04	0.02	7.4E-03	-0.43	-0.10	No
rs2294239	G	-0.24	0.04	3.5E-08	-0.17	0.04	4.4E-06	-0.02	0.01	4.6E-02	-0.21	-0.07	No
rs2301597	T	0.35	0.04	5.8E-16	0.29	0.04	4.9E-14	0.00	0.01	9.8E-01	0.32	0.07	No
rs2306363	T	-0.34	0.05	9.9E-11	-0.38	0.05	1.2E-16	-0.05	0.01	2.0E-05	-0.36	0.04	No
rs2311411	A	-0.13	0.04	2.1E-03	-0.23	0.04	3.0E-09	-0.03	0.01	8.7E-03	-0.18	0.09	No
rs2478531	C	0.33	0.04	5.5E-14	0.24	0.04	1.4E-10	0.02	0.03	4.7E-01	0.29	0.08	No
rs2478981	T	0.19	0.05	3.7E-05	0.29	0.04	2.6E-13	0.01	0.01	4.1E-01	0.24	-0.10	No
rs2493296	T	0.33	0.06	2.0E-07	0.39	0.05	4.7E-13	0.06	0.01	1.4E-04	0.36	-0.07	No
rs2521501	T	0.48	0.05	8.6E-25	0.50	0.04	3.2E-36	0.06	0.01	5.0E-08	0.49	-0.03	No
rs2627308	A	0.23	0.04	1.1E-07	0.32	0.04	1.3E-17	0.00	0.01	6.6E-01	0.27	-0.09	No
rs262986	A	-0.15	0.04	3.4E-04	-0.23	0.04	2.3E-09	0.00	0.01	8.7E-01	-0.19	0.07	No
rs2644128	C	-0.24	0.04	1.5E-08	-0.11	0.04	4.4E-03	-0.02	0.01	2.3E-02	-0.18	-0.14	No
rs268263	T	-0.53	0.05	1.2E-25	-0.33	0.04	4.3E-14	-0.04	0.01	1.2E-04	-0.43	-0.19	No
rs2760061	A	0.23	0.04	1.5E-07	0.26	0.04	1.1E-11	0.00	0.01	9.2E-01	0.24	-0.03	No
rs2823139	A	0.05	0.05	2.4E-01	0.24	0.04	1.3E-09	0.03	0.01	6.4E-04	0.15	-0.19	No
rs28416181	G	-0.36	0.05	6.6E-13	-0.24	0.04	4.3E-08	-0.02	0.01	6.3E-02	-0.30	-0.12	No
rs28572357	C	0.23	0.04	1.5E-07	0.23	0.04	2.7E-09	0.01	0.01	1.2E-01	0.23	0.00	No
rs286749	T	-0.12	0.06	3.1E-02	-0.29	0.05	6.7E-09	-0.04	0.01	3.6E-04	-0.21	0.17	No
rs28866311	G	0.33	0.04	7.0E-15	0.24	0.04	2.3E-10	0.01	0.01	1.3E-01	0.29	0.10	No
rs2947411	A	-0.35	0.06	7.2E-10	-0.18	0.05	1.8E-04	-0.03	0.01	4.1E-03	-0.27	-0.16	No
rs311443	G	-0.11	0.05	2.7E-02	-0.24	0.04	1.2E-08	-0.01	0.01	2.7E-01	-0.17	0.13	No
rs3118905	A	0.28	0.05	2.7E-09	0.13	0.04	1.3E-03	0.01	0.01	2.1E-01	0.21	0.15	No
rs3218036	A	0.24	0.05	1.3E-07	0.26	0.04	1.5E-10	-0.02	0.01	4.0E-02	0.25	-0.01	No
rs34148132	T	-0.24	0.05	2.8E-07	-0.24	0.04	7.5E-09	-0.05	0.01	3.8E-06	-0.24	0.00	No
rs34394882	T	0.28	0.05	4.3E-09	0.16	0.04	9.1E-05	-0.01	0.01	5.7E-01	0.22	0.12	No
rs351365	T	-0.31	0.05	3.9E-10	-0.40	0.04	1.4E-20	0.00	0.01	9.4E-01	-0.36	0.09	No
rs35444	G	-0.27	0.04	4.4E-10	-0.43	0.04	2.3E-29	0.00	0.01	8.5E-01	-0.35	0.16	No
rs35681682	C	-0.14	0.04	1.4E-03	-0.22	0.04	2.7E-09	-0.02	0.01	1.6E-02	-0.18	0.09	No
rs35942721	T	-0.27	0.05	1.7E-08	-0.27	0.04	1.0E-10	-0.03	0.01	1.7E-02	-0.27	0.00	No
rs3746038	T	-0.17	0.05	1.7E-03	-0.28	0.05	1.6E-09	0.00	0.01	9.6E-01	-0.23	0.11	No
rs3764769	T	-0.13	0.05	7.8E-03	-0.35	0.04	9.0E-17	0.00	0.01	9.6E-01	-0.24	0.22	Yes
rs3803266	G	0.19	0.05	1.3E-04	0.27	0.04	1.5E-09	-0.01	0.01	5.5E-01	0.23	-0.07	No

rs3821843	G	-0.17	0.05	2.0E-04	-0.34	0.04	5.3E-17	-0.01	0.01	4.6E-01	-0.26	0.17	No
rs389883	G	-0.34	0.05	5.2E-14	-0.26	0.04	6.6E-11	-0.03	0.01	2.2E-02	-0.30	-0.08	No
rs3918226	T	0.68	0.08	2.2E-17	0.65	0.07	4.9E-21	0.13	0.02	1.7E-09	0.66	0.02	No
rs3936510	T	0.33	0.05	8.8E-10	0.13	0.05	7.0E-03	0.04	0.01	4.7E-04	0.23	0.20	Yes
rs4076877	T	-0.59	0.10	1.3E-09	-0.19	0.09	2.6E-02	-0.07	0.03	4.4E-02	-0.39	-0.40	Yes
rs4147111	C	-0.50	0.08	3.1E-09	-0.24	0.07	1.2E-03	-0.07	0.02	3.6E-04	-0.37	-0.26	Yes
rs4362428	A	-0.27	0.04	6.3E-10	-0.25	0.04	4.6E-11	0.00	0.01	7.6E-01	-0.26	-0.02	No
rs4428270	C	0.26	0.04	3.4E-09	0.20	0.04	1.2E-07	0.02	0.01	2.3E-02	0.23	0.06	No
rs4473575	G	-0.11	0.08	1.7E-01	-0.39	0.07	3.3E-08	0.03	0.02	1.6E-01	-0.25	0.28	Yes
rs4588930	A	-0.24	0.04	1.9E-08	-0.06	0.04	9.2E-02	-0.01	0.01	5.4E-01	-0.15	-0.18	No
rs4712656	C	0.27	0.04	4.2E-10	0.18	0.04	2.0E-06	0.01	0.01	3.9E-01	0.22	0.09	No
rs483071	C	-0.21	0.04	3.8E-06	-0.24	0.04	5.9E-10	0.00	0.01	6.8E-01	-0.22	0.03	No
rs4873492	T	0.27	0.06	3.8E-06	0.33	0.05	2.7E-11	0.01	0.01	2.5E-01	0.30	-0.07	No
rs488834	C	0.26	0.05	2.0E-07	0.36	0.04	2.9E-16	0.01	0.01	4.4E-01	0.31	-0.10	No
rs4899396	T	0.04	0.04	3.6E-01	0.22	0.04	7.6E-09	0.01	0.01	6.0E-01	0.13	-0.18	No
rs4923914	T	0.16	0.05	3.6E-04	0.27	0.04	2.5E-11	-0.02	0.01	8.2E-02	0.21	-0.10	No
rs507666	A	-0.20	0.06	2.4E-04	-0.26	0.05	3.6E-08	0.08	0.01	1.6E-11	-0.23	0.06	No
rs5418	G	-0.21	0.04	1.1E-06	-0.29	0.04	2.2E-14	0.01	0.01	1.4E-01	-0.25	0.08	No
rs55646464	T	-0.27	0.05	1.0E-08	-0.32	0.04	2.9E-15	-0.01	0.01	3.9E-01	-0.30	0.05	No
rs55733296	A	0.04	0.11	7.1E-01	0.60	0.10	6.5E-10	0.07	0.04	1.4E-01	0.32	-0.56	Yes
rs56179563	A	-0.30	0.04	9.0E-12	-0.17	0.04	8.2E-06	-0.07	0.01	7.5E-10	-0.24	-0.13	No
rs56313611	T	-0.39	0.06	3.3E-10	-0.22	0.05	3.1E-05	-0.06	0.01	1.1E-05	-0.31	-0.16	No
rs56352102	T	0.39	0.06	1.8E-12	0.41	0.05	1.2E-17	0.03	0.01	1.4E-02	0.40	-0.02	No
rs569550	G	0.31	0.04	4.7E-12	0.45	0.04	3.1E-32	0.02	0.01	1.4E-01	0.38	-0.15	No
rs57139556	G	-0.59	0.08	8.6E-13	-0.60	0.07	1.6E-16	-0.05	0.02	3.5E-03	-0.60	0.00	No
rs57786342	A	0.33	0.05	3.6E-10	0.11	0.05	2.3E-02	0.02	0.01	2.1E-01	0.22	0.23	Yes
rs6031431	G	0.13	0.04	2.1E-03	0.24	0.04	3.1E-10	0.00	0.01	8.1E-01	0.19	-0.10	No
rs604723	T	-0.60	0.05	5.2E-36	-0.55	0.04	2.4E-39	-0.03	0.01	1.2E-03	-0.58	-0.05	No
rs6108171	T	-0.25	0.05	3.5E-07	-0.26	0.04	1.6E-09	0.00	0.01	7.8E-01	-0.26	0.01	No
rs6108787	G	0.36	0.04	7.4E-17	0.40	0.04	9.8E-27	0.02	0.01	1.6E-02	0.38	-0.04	No
rs62036942	C	-0.39	0.06	4.3E-10	-0.23	0.05	2.7E-05	0.00	0.01	8.0E-01	-0.31	-0.16	No
rs62390667	G	0.24	0.04	3.8E-08	0.32	0.04	1.0E-16	0.03	0.01	2.7E-03	0.28	-0.08	No
rs62447769	A	0.70	0.13	4.6E-08	0.24	0.11	2.9E-02	0.02	0.03	3.6E-01	0.47	0.45	Yes
rs6433891	G	-0.11	0.05	2.1E-02	-0.27	0.04	5.9E-11	-0.02	0.01	6.8E-02	-0.19	0.16	No
rs6438857	C	-0.25	0.04	8.2E-09	-0.19	0.04	4.1E-07	-0.01	0.01	2.5E-01	-0.22	-0.06	No
rs645040	G	-0.12	0.05	1.5E-02	-0.25	0.04	2.0E-08	-0.04	0.01	6.0E-04	-0.19	0.13	No
rs6490019	A	-0.18	0.04	5.8E-05	-0.28	0.04	6.4E-13	-0.01	0.01	5.1E-01	-0.23	0.10	No
rs66682451	G	-0.21	0.05	1.5E-05	-0.31	0.04	1.0E-13	-0.02	0.01	3.3E-02	-0.26	0.10	No
rs6697193	T	-0.14	0.06	1.5E-02	-0.34	0.05	2.2E-11	0.01	0.02	4.4E-01	-0.24	0.20	Yes
rs6777317	A	0.09	0.05	7.2E-02	0.23	0.04	3.7E-08	0.00	0.01	9.6E-01	0.16	-0.14	No
rs6798940	A	-0.25	0.04	8.4E-09	-0.13	0.04	6.0E-04	0.00	0.01	8.5E-01	-0.19	-0.12	No
rs6815273	A	-0.16	0.04	3.1E-04	-0.23	0.04	1.8E-09	-0.01	0.01	2.2E-01	-0.19	0.07	No
rs6827655	G	0.11	0.05	2.5E-02	0.24	0.04	1.8E-08	0.03	0.01	3.4E-03	0.17	-0.13	No
rs6928622	C	0.11	0.04	9.4E-03	0.21	0.04	3.6E-08	0.00	0.01	5.9E-01	0.16	-0.09	No
rs6961048	G	0.22	0.07	2.4E-03	0.41	0.06	3.7E-11	0.01	0.02	3.9E-01	0.31	-0.20	No
rs6963105	A	-0.19	0.04	7.1E-06	-0.22	0.04	3.5E-09	-0.02	0.01	1.3E-01	-0.21	0.03	No
rs6990531	G	-0.35	0.05	2.6E-12	-0.19	0.04	1.8E-05	0.00	0.01	9.1E-01	-0.27	-0.16	No
rs7107356	A	-0.25	0.04	4.4E-09	-0.31	0.04	4.0E-17	-0.01	0.01	5.0E-01	-0.28	0.06	No
rs7116280	C	0.20	0.05	2.9E-05	0.24	0.04	7.7E-09	0.02	0.01	1.2E-01	0.22	-0.04	No
rs7121365	A	0.44	0.07	8.8E-12	0.20	0.06	4.4E-04	0.02	0.01	8.5E-02	0.32	0.24	Yes
rs71326977	A	-0.06	0.06	2.9E-01	-0.31	0.05	7.8E-10	-0.03	0.01	4.5E-03	-0.18	0.25	Yes
rs71508634	T	-0.26	0.05	4.0E-08	-0.06	0.04	1.4E-01	0.00	0.01	6.4E-01	-0.16	-0.20	No
rs71654213	T	-0.30	0.04	1.6E-11	-0.13	0.04	1.1E-03	0.02	0.01	3.8E-02	-0.21	-0.17	No
rs7223364	T	0.29	0.07	7.1E-06	0.37	0.06	7.3E-11	0.01	0.01	3.2E-01	0.33	-0.08	No
rs72654647	A	0.33	0.05	5.7E-11	0.20	0.04	3.3E-06	0.03	0.01	1.6E-02	0.27	0.12	No
rs72677850	A	-1.22	0.16	5.1E-14	-0.80	0.14	9.8E-09	-0.07	0.04	7.3E-02	-1.01	-0.41	Yes
rs72792829	T	-0.21	0.05	6.4E-05	-0.27	0.05	3.8E-09	-0.01	0.01	2.1E-01	-0.24	0.06	No
rs72811742	T	-0.07	0.04	1.3E-01	-0.22	0.04	2.0E-08	-0.01	0.01	2.8E-01	-0.14	0.15	No
rs72831343	G	-0.55	0.06	1.1E-19	-0.61	0.05	3.6E-31	-0.02	0.02	2.8E-01	-0.58	0.06	No
rs72844588	A	0.21	0.06	5.0E-04	0.29	0.05	1.8E-08	0.01	0.02	3.2E-01	0.25	-0.09	No
rs72854462	G	0.29	0.05	7.2E-09	0.25	0.04	1.0E-08	-0.03	0.01	2.6E-02	0.27	0.04	No
rs72936986	C	-0.24	0.05	4.3E-07	-0.25	0.04	2.0E-09	0.01	0.01	5.3E-01	-0.25	0.01	No
rs72976751	T	0.36	0.06	7.1E-09	0.29	0.05	8.6E-08	0.01	0.01	5.3E-01	0.32	0.07	No

rs7302981	A	0.37	0.04	3.2E-17	0.30	0.04	1.5E-14	0.00	0.01	9.8E-01	0.34	0.08	No
rs73030267	C	-0.26	0.09	2.8E-03	-0.47	0.08	3.1E-10	-0.02	0.02	1.4E-01	-0.37	0.21	Yes
rs73033340	G	-0.58	0.12	2.1E-06	-0.82	0.11	1.4E-14	0.01	0.03	7.6E-01	-0.70	0.24	Yes
rs73046792	A	-0.35	0.06	1.1E-09	-0.33	0.05	3.0E-11	-0.02	0.02	3.2E-01	-0.34	-0.02	No
rs7306710	T	-0.07	0.04	1.3E-01	-0.22	0.04	8.4E-09	-0.02	0.01	3.0E-02	-0.14	0.15	No
rs73075659	G	-0.41	0.05	1.1E-19	-0.30	0.04	4.6E-14	-0.01	0.01	5.6E-01	-0.35	-0.11	No
rs7310615	C	0.52	0.04	9.8E-33	0.53	0.04	1.5E-45	0.06	0.01	1.7E-09	0.52	-0.02	No
rs73234219	T	0.24	0.04	2.4E-08	0.07	0.04	7.9E-02	-0.01	0.01	1.7E-01	0.16	0.18	No
rs73306860	A	0.60	0.07	4.7E-18	0.61	0.06	1.2E-23	0.04	0.02	4.7E-03	0.60	0.00	No
rs7340705	C	0.19	0.05	3.7E-05	0.24	0.04	1.4E-09	0.01	0.01	5.6E-01	0.22	-0.05	No
rs74233809	C	-0.77	0.08	4.5E-22	-0.56	0.07	1.4E-15	-0.07	0.01	4.1E-07	-0.67	-0.21	Yes
rs743509	C	0.09	0.05	5.8E-02	0.23	0.04	1.2E-08	0.00	0.01	8.1E-01	0.16	-0.14	No
rs7463212	T	0.17	0.04	1.1E-04	0.29	0.04	4.1E-15	0.00	0.01	6.1E-01	0.23	-0.13	No
rs74661587	G	0.53	0.06	1.1E-16	0.25	0.06	4.2E-06	0.02	0.01	1.8E-01	0.39	0.27	Yes
rs747423	T	0.27	0.05	2.4E-08	0.18	0.04	4.6E-05	0.01	0.01	6.0E-01	0.22	0.10	No
rs7484151	A	-0.34	0.06	3.4E-08	-0.34	0.05	2.2E-10	0.03	0.01	3.8E-02	-0.34	0.00	No
rs748676	A	0.54	0.08	3.3E-11	0.19	0.07	7.6E-03	0.01	0.01	5.9E-01	0.37	0.35	Yes
rs751984	C	-0.44	0.07	4.9E-11	-0.48	0.06	4.0E-16	-0.02	0.01	9.9E-02	-0.46	0.04	No
rs76452347	T	-0.25	0.06	6.9E-06	-0.30	0.05	3.7E-10	0.01	0.01	6.6E-01	-0.27	0.05	No
rs7675258	G	0.11	0.04	9.2E-03	0.22	0.04	4.3E-09	0.01	0.01	2.2E-01	0.17	-0.11	No
rs7763581	G	-0.08	0.04	6.5E-02	-0.22	0.04	6.2E-09	0.01	0.01	2.6E-01	-0.15	0.14	No
rs778124	A	0.06	0.04	1.6E-01	0.23	0.04	2.0E-09	0.02	0.01	1.7E-02	0.15	-0.17	No
rs77924615	A	-0.35	0.05	1.9E-10	-0.45	0.05	1.7E-21	0.00	0.01	7.0E-01	-0.40	0.11	No
rs7810386	A	0.14	0.04	1.3E-03	0.26	0.04	1.3E-11	0.02	0.01	1.2E-02	0.20	-0.12	No
rs78203196	C	-0.30	0.07	1.1E-05	-0.37	0.06	4.0E-10	0.01	0.01	5.0E-01	-0.33	0.07	No
rs7831859	C	-0.21	0.04	1.6E-06	-0.21	0.04	3.6E-08	-0.01	0.01	5.4E-01	-0.21	0.00	No
rs7838131	G	0.30	0.04	3.7E-12	0.34	0.04	1.9E-19	-0.01	0.01	6.1E-01	0.32	-0.04	No
rs7845053	A	-0.26	0.04	5.4E-09	-0.01	0.04	7.0E-01	0.01	0.01	3.1E-01	-0.14	-0.24	Yes
rs79023617	T	-0.11	0.04	1.3E-02	-0.33	0.04	1.5E-17	0.00	0.01	8.5E-01	-0.22	0.22	Yes
rs7911644	T	0.23	0.05	5.8E-07	0.33	0.04	1.2E-16	0.01	0.01	1.8E-01	0.28	-0.10	No
rs7938856	C	0.09	0.05	5.9E-02	0.23	0.04	2.9E-08	-0.01	0.01	3.9E-01	0.16	-0.14	No
rs79780963	T	-0.77	0.08	1.5E-21	-0.59	0.07	7.4E-17	-0.08	0.01	7.5E-08	-0.68	-0.18	No
rs7989142	G	0.18	0.05	2.9E-04	0.32	0.04	2.3E-13	0.01	0.01	5.8E-01	0.25	-0.14	No
rs79997166	C	0.54	0.09	2.6E-09	0.12	0.08	1.3E-01	0.01	0.03	6.6E-01	0.33	0.42	Yes
rs8044992	C	-0.29	0.05	1.9E-09	-0.16	0.04	6.7E-05	-0.01	0.01	2.3E-01	-0.22	-0.12	No
rs8059962	T	-0.18	0.04	4.1E-05	-0.21	0.04	1.8E-08	0.00	0.01	9.5E-01	-0.20	0.04	No
rs8070460	C	-0.27	0.04	4.9E-10	-0.11	0.04	2.4E-03	-0.04	0.01	2.1E-05	-0.19	-0.15	No
rs8112983	T	-0.13	0.04	3.1E-03	-0.20	0.04	5.0E-08	0.00	0.01	7.4E-01	-0.17	0.08	No
rs897264	T	0.00	0.05	9.4E-01	-0.27	0.05	1.1E-09	-0.01	0.01	5.9E-01	-0.14	0.28	Yes
rs9314268	C	0.14	0.05	4.0E-03	0.25	0.04	8.9E-09	-0.01	0.01	5.5E-01	0.20	-0.11	No
rs9375463	C	0.33	0.04	1.9E-14	0.48	0.04	1.4E-37	0.01	0.01	4.6E-01	0.41	-0.15	No
rs9394951	C	0.22	0.04	2.7E-07	0.21	0.04	4.5E-08	0.01	0.01	1.6E-01	0.21	0.02	No
rs9482120	C	-0.14	0.04	1.2E-03	-0.25	0.04	7.9E-11	0.00	0.01	6.1E-01	-0.20	0.11	No
rs966791	T	-0.12	0.04	5.8E-03	-0.27	0.04	3.9E-13	-0.01	0.01	3.2E-01	-0.20	0.15	No
rs9719973	A	0.33	0.04	8.7E-14	0.16	0.04	3.6E-05	0.01	0.01	2.4E-01	0.24	0.17	No
rs990902	C	-0.10	0.04	2.4E-02	-0.21	0.04	2.4E-08	-0.01	0.01	3.8E-01	-0.16	0.11	No
rs9927137	G	0.19	0.04	1.2E-05	0.24	0.04	1.1E-10	0.00	0.01	9.8E-01	0.22	-0.05	No

Table S5. Instruments and genetic association estimates for Model 2 of the multivariable Mendelian randomization analysis. GX: genetic association estimate with MAP ≤55 years (mmHg); GY: genetic association estimate with coronary artery disease (log odds ratio); GZ: genetic association estimates with MAP >55 years (mmHg); SE: standard error; SNP: single-nucleotide polymorphism.

SNP	Effect Allele	GX	GX_SE	GX_P	GZ	GZ_SE	GZ_P	GY	GY_SE	GY_P
rs1000423	C	-0.455	0.115	7.76E-05	-0.228	0.100	2.28E-02	-0.016	0.034	6.45E-01
rs1010064	C	-0.501	0.083	1.65E-09	-0.632	0.072	2.18E-18	-0.017	0.024	4.91E-01
rs10119435	A	0.267	0.059	5.28E-06	0.309	0.051	1.38E-09	0.010	0.017	5.76E-01
rs10158537	G	-0.425	0.059	6.66E-13	-0.443	0.052	9.88E-18	0.024	0.017	1.61E-01
rs10226118	C	-0.824	0.109	4.53E-14	-0.496	0.096	2.20E-07	-0.001	0.032	9.74E-01
rs10248237	A	-0.819	0.110	8.92E-14	-0.531	0.096	3.58E-08	0.001	0.032	9.87E-01
rs10265221	C	0.122	0.059	3.74E-02	0.239	0.051	2.89E-06	-0.021	0.017	2.13E-01
rs10409243	C	-0.531	0.067	2.44E-15	-0.368	0.058	2.51E-10	-0.038	0.020	5.27E-02
rs10468291	C	0.193	0.071	6.23E-03	0.269	0.061	1.17E-05	-0.003	0.021	8.77E-01
rs10777213	G	0.149	0.062	1.68E-02	0.325	0.054	2.55E-09	0.015	0.018	4.18E-01
rs10852034	T	-0.523	0.064	4.62E-16	-0.459	0.056	1.68E-16	-0.014	0.019	4.58E-01
rs10858071	A	0.124	0.061	4.21E-02	0.222	0.053	2.46E-05	-0.049	0.018	5.98E-03
rs10876531	C	0.030	0.060	6.16E-01	0.194	0.052	1.89E-04	-0.021	0.017	2.22E-01
rs10900127	C	0.098	0.062	1.13E-01	0.229	0.054	2.24E-05	0.007	0.018	6.91E-01
rs10993958	A	0.301	0.060	6.63E-07	0.519	0.053	5.25E-23	0.034	0.018	5.67E-02
rs10995307	T	-0.385	0.093	3.19E-05	-0.407	0.080	4.17E-07	-0.036	0.027	1.91E-01
rs11070245	T	-0.322	0.072	9.05E-06	-0.387	0.063	8.80E-10	-0.024	0.021	2.69E-01
rs11072508	C	0.077	0.067	2.45E-01	0.272	0.058	2.41E-06	-0.003	0.019	8.73E-01
rs1114348	A	-0.598	0.066	1.53E-19	-0.616	0.057	7.98E-27	-0.057	0.020	3.79E-03
rs11187838	A	-0.138	0.066	3.60E-02	-0.336	0.057	3.36E-09	-0.039	0.019	4.53E-02
rs113044050	T	0.178	0.065	6.41E-03	0.183	0.057	1.29E-03	0.011	0.019	5.78E-01
rs113230003	A	0.374	0.089	2.54E-05	0.281	0.078	3.14E-04	0.075	0.026	3.34E-03
rs1133400	G	0.386	0.076	3.65E-07	0.393	0.066	2.30E-09	0.045	0.022	3.99E-02
rs113458760	G	0.340	0.062	4.76E-08	0.190	0.054	4.28E-04	0.009	0.018	6.11E-01
rs113695818	T	-0.235	0.065	2.91E-04	-0.198	0.056	4.19E-04	-0.034	0.019	7.68E-02
rs115262049	T	0.263	0.067	9.75E-05	0.291	0.059	6.50E-07	0.006	0.020	7.55E-01
rs11642015	T	0.411	0.115	3.36E-04	0.452	0.101	6.97E-06	0.075	0.033	2.31E-02
rs11669915	G	-0.043	0.153	7.76E-01	0.521	0.134	9.88E-05	0.043	0.044	3.35E-01
rs11676040	C	-0.287	0.059	9.57E-07	-0.304	0.051	2.48E-09	0.014	0.017	4.27E-01
rs11749673	G	-0.363	0.084	1.50E-05	-0.364	0.073	5.16E-07	-0.026	0.025	2.95E-01
rs1175651	T	-0.106	0.059	7.22E-02	-0.208	0.051	5.03E-05	0.002	0.017	8.91E-01
rs11760498	A	-0.547	0.078	2.20E-12	-0.585	0.068	8.85E-18	0.024	0.023	3.00E-01
rs11774829	A	0.337	0.059	9.76E-09	0.122	0.051	1.70E-02	0.005	0.017	7.51E-01
rs11821781	G	0.497	0.059	4.94E-17	0.466	0.051	1.22E-19	0.088	0.017	3.13E-07
rs11915142	A	-0.318	0.071	7.76E-06	-0.165	0.062	7.39E-03	-0.038	0.021	6.76E-02
rs12057453	T	-0.255	0.060	2.35E-05	-0.439	0.052	4.83E-17	0.020	0.018	2.60E-01
rs12137438	C	-0.155	0.060	1.04E-02	-0.316	0.053	2.31E-09	0.007	0.018	7.10E-01
rs12194642	A	-0.097	0.064	1.27E-01	-0.338	0.055	1.02E-09	-0.012	0.019	5.31E-01
rs12216886	G	-0.244	0.076	1.43E-03	-0.319	0.066	1.26E-06	0.002	0.022	9.11E-01
rs12258967	G	-0.411	0.062	3.26E-11	-0.385	0.054	8.87E-13	-0.017	0.018	3.60E-01
rs1229984	T	0.429	0.202	3.32E-02	0.936	0.178	1.44E-07	-0.028	0.060	6.43E-01
rs12567136	T	0.417	0.061	5.55E-12	0.307	0.053	6.60E-09	-0.012	0.018	4.85E-01
rs12627514	G	-0.159	0.065	1.41E-02	-0.244	0.057	1.57E-05	-0.015	0.019	4.46E-01
rs12643599	G	-0.220	0.061	3.30E-04	-0.279	0.053	1.64E-07	0.000	0.018	9.92E-01
rs12656497	T	0.216	0.069	1.78E-03	0.339	0.060	1.67E-08	-0.018	0.020	3.77E-01
rs12693302	G	0.133	0.070	5.55E-02	0.239	0.060	7.65E-05	0.010	0.020	6.16E-01
rs1275978	C	0.280	0.065	1.87E-05	0.120	0.057	3.56E-02	0.026	0.019	1.77E-01
rs12978472	G	0.539	0.112	1.44E-06	0.191	0.097	4.92E-02	0.019	0.033	5.57E-01
rs12983032	A	-0.272	0.085	1.39E-03	-0.368	0.074	6.00E-07	-0.041	0.025	1.06E-01
rs13107325	T	-1.000	0.222	6.50E-06	-0.939	0.190	7.83E-07	-0.063	0.066	3.44E-01
rs13121442	T	0.251	0.073	5.84E-04	0.101	0.064	1.12E-01	0.004	0.021	8.69E-01
rs13125101	A	-0.007	0.059	9.08E-01	0.186	0.052	3.25E-04	-0.013	0.017	4.47E-01
rs13227860	A	0.472	0.064	1.05E-13	0.483	0.055	1.13E-18	0.072	0.018	8.38E-05
rs13324341	T	-0.141	0.060	1.79E-02	-0.198	0.052	1.36E-04	-0.019	0.017	2.85E-01
rs1436049	G	-0.212	0.059	3.26E-04	-0.209	0.051	4.61E-05	-0.036	0.017	3.88E-02

rs1436138	G	0.333	0.059	1.44E-08	0.242	0.051	2.13E-06	0.017	0.017	3.13E-01
rs1472467	G	0.174	0.062	5.21E-03	0.298	0.054	4.25E-08	0.037	0.018	4.10E-02
rs1487629	A	0.523	0.063	1.00E-16	0.232	0.055	2.06E-05	0.019	0.018	2.98E-01
rs1492027	G	0.231	0.059	8.78E-05	0.261	0.051	3.57E-07	0.009	0.017	5.91E-01
rs150857355	C	0.179	0.103	8.39E-02	0.426	0.090	2.27E-06	-0.016	0.030	6.00E-01
rs1544861	T	-0.471	0.085	2.62E-08	-0.263	0.074	3.62E-04	-0.022	0.025	3.80E-01
rs164101	G	-0.189	0.060	1.58E-03	-0.207	0.052	6.88E-05	-0.014	0.018	4.33E-01
rs1687318	T	-0.421	0.074	1.33E-08	-0.481	0.065	8.71E-14	-0.010	0.022	6.62E-01
rs16895971	C	-0.339	0.065	1.83E-07	-0.167	0.056	3.07E-03	0.012	0.019	5.33E-01
rs17249754	A	0.212	0.059	3.35E-04	0.312	0.051	1.12E-09	0.015	0.017	3.71E-01
rs17257695	G	0.080	0.060	1.80E-01	0.168	0.052	1.13E-03	-0.012	0.017	4.91E-01
rs17517959	C	0.311	0.060	2.15E-07	0.061	0.052	2.42E-01	0.020	0.017	2.63E-01
rs17637472	A	-0.100	0.067	1.36E-01	-0.252	0.058	1.43E-05	-0.001	0.020	9.54E-01
rs17717829	C	-0.246	0.060	3.78E-05	-0.182	0.052	4.64E-04	0.000	0.017	9.85E-01
rs17762	A	-0.125	0.061	4.10E-02	-0.276	0.053	2.33E-07	-0.048	0.018	8.72E-03
rs1801253	G	0.342	0.067	3.63E-07	0.130	0.059	2.76E-02	0.032	0.020	9.84E-02
rs187680068	G	-0.214	0.062	5.06E-04	-0.199	0.054	1.96E-04	-0.011	0.018	5.40E-01
rs1896326	A	-0.190	0.059	1.33E-03	-0.244	0.052	2.29E-06	-0.007	0.017	6.79E-01
rs1958603	A	0.262	0.090	3.43E-03	0.334	0.078	1.96E-05	0.007	0.026	8.00E-01
rs2014590	T	0.391	0.060	5.19E-11	0.324	0.052	3.99E-10	0.007	0.017	7.00E-01
rs2032451	T	0.236	0.060	8.73E-05	0.169	0.053	1.26E-03	0.033	0.018	6.05E-02
rs2050905	G	-0.155	0.066	1.96E-02	-0.304	0.058	1.46E-07	-0.015	0.020	4.32E-01
rs2067832	G	-0.287	0.059	1.16E-06	-0.158	0.051	2.06E-03	-0.016	0.017	3.42E-01
rs2071265	C	0.376	0.064	5.69E-09	0.139	0.056	1.28E-02	-0.003	0.019	8.68E-01
rs2073641	A	-0.097	0.074	1.88E-01	-0.263	0.064	3.80E-05	-0.008	0.021	6.99E-01
rs2087319	A	-0.230	0.062	1.99E-04	-0.189	0.054	4.33E-04	0.008	0.018	6.68E-01
rs2165301	C	-0.662	0.089	7.35E-14	-0.675	0.076	7.51E-19	0.009	0.026	7.38E-01
rs2178270	C	0.165	0.061	6.80E-03	0.212	0.053	5.82E-05	0.002	0.018	9.02E-01
rs2242338	C	-0.291	0.067	1.61E-05	-0.216	0.058	2.22E-04	-0.042	0.020	3.28E-02
rs2294239	G	0.221	0.063	4.20E-04	0.254	0.054	2.95E-06	0.018	0.018	3.17E-01
rs2301597	T	0.250	0.060	3.17E-05	0.259	0.052	6.92E-07	-0.023	0.018	1.89E-01
rs2306363	T	0.329	0.067	1.01E-06	0.200	0.059	6.45E-04	0.053	0.019	6.90E-03
rs2311411	A	-0.140	0.059	1.77E-02	-0.215	0.051	2.67E-05	-0.010	0.017	5.48E-01
rs2478531	C	-0.383	0.079	1.25E-06	-0.314	0.069	4.95E-06	0.000	0.023	9.95E-01
rs2478981	T	0.328	0.086	1.32E-04	0.406	0.074	4.64E-08	0.023	0.025	3.49E-01
rs2493296	T	0.177	0.060	3.37E-03	0.278	0.053	1.29E-07	0.005	0.018	7.94E-01
rs2521501	T	0.259	0.072	3.28E-04	0.287	0.063	4.70E-06	0.023	0.021	2.68E-01
rs2627308	A	-0.337	0.068	6.47E-07	-0.461	0.059	5.60E-15	0.004	0.020	8.32E-01
rs262986	A	1.266	0.201	3.30E-10	0.520	0.178	3.41E-03	0.086	0.059	1.45E-01
rs2644128	C	0.298	0.069	1.63E-05	0.322	0.060	1.01E-07	0.047	0.020	1.94E-02
rs268263	T	-0.599	0.080	5.24E-14	-0.670	0.069	3.03E-22	-0.018	0.023	4.46E-01
rs2760061	A	0.234	0.060	9.21E-05	0.113	0.052	3.00E-02	0.017	0.017	3.40E-01
rs2823139	A	-0.293	0.059	6.54E-07	-0.206	0.051	6.28E-05	-0.001	0.017	9.61E-01
rs28416181	G	0.147	0.061	1.55E-02	0.259	0.053	9.01E-07	0.009	0.018	5.99E-01
rs28572357	C	0.180	0.059	2.46E-03	0.308	0.052	2.51E-09	-0.017	0.017	3.18E-01
rs286749	T	0.358	0.060	2.31E-09	0.164	0.052	1.60E-03	0.027	0.017	1.24E-01
rs28866311	G	0.250	0.064	9.41E-05	0.289	0.056	1.98E-07	-0.053	0.019	4.95E-03
rs2947411	A	-0.158	0.081	5.10E-02	-0.349	0.070	6.71E-07	0.033	0.023	1.63E-01
rs311443	G	0.321	0.068	2.69E-06	0.262	0.059	1.01E-05	-0.009	0.020	6.53E-01
rs3118905	A	-0.109	0.066	1.00E-01	-0.224	0.058	1.03E-04	-0.001	0.019	9.45E-01
rs3218036	A	-0.318	0.061	1.67E-07	-0.178	0.053	8.00E-04	0.018	0.018	3.03E-01
rs34148132	T	-0.001	0.061	9.93E-01	0.187	0.053	4.14E-04	0.006	0.018	7.46E-01
rs34394882	T	-0.280	0.069	4.31E-05	-0.164	0.059	5.47E-03	-0.030	0.020	1.32E-01
rs351365	T	0.370	0.059	3.23E-10	0.328	0.051	1.60E-10	0.008	0.017	6.52E-01
rs35444	G	0.140	0.059	1.84E-02	0.264	0.051	2.76E-07	0.029	0.017	9.42E-02
rs35681682	C	-0.439	0.085	2.16E-07	-0.216	0.074	3.42E-03	-0.047	0.025	6.20E-02
rs35942721	T	0.745	0.095	3.25E-15	0.625	0.083	3.71E-14	0.054	0.027	4.61E-02
rs3746038	T	0.184	0.062	2.89E-03	0.321	0.054	2.35E-09	0.040	0.018	2.73E-02
rs3764769	T	0.023	0.062	7.11E-01	0.223	0.054	4.05E-05	0.033	0.018	6.63E-02
rs3803266	G	-0.080	0.078	3.09E-01	-0.279	0.068	4.09E-05	-0.029	0.023	2.10E-01
rs3821843	G	0.362	0.065	2.80E-08	0.229	0.057	5.11E-05	0.020	0.019	2.84E-01
rs389883	G	-0.282	0.059	2.07E-06	-0.245	0.052	2.41E-06	-0.017	0.017	3.19E-01
rs3918226	T	-0.400	0.077	2.44E-07	-0.169	0.067	1.18E-02	0.031	0.022	1.70E-01

rs3936510	T	-0.028	0.061	6.52E-01	-0.205	0.053	1.15E-04	-0.003	0.018	8.72E-01
rs4076877	T	-0.081	0.060	1.75E-01	-0.204	0.052	8.36E-05	-0.045	0.017	1.02E-02
rs4147111	C	-0.100	0.058	8.80E-02	-0.268	0.051	1.41E-07	-0.007	0.017	6.65E-01
rs4362428	A	0.123	0.064	5.46E-02	0.353	0.056	2.35E-10	0.018	0.019	3.49E-01
rs4428270	C	0.250	0.082	2.16E-03	0.334	0.071	2.75E-06	0.014	0.024	5.61E-01
rs4473575	G	0.362	0.068	1.02E-07	0.291	0.059	8.27E-07	-0.023	0.020	2.45E-01
rs4588930	A	-0.251	0.066	1.30E-04	-0.245	0.057	1.65E-05	-0.014	0.019	4.70E-01
rs4712656	C	0.429	0.124	5.28E-04	0.128	0.107	2.31E-01	0.020	0.036	5.66E-01
rs483071	C	-0.454	0.069	3.86E-11	-0.263	0.060	1.15E-05	-0.028	0.020	1.70E-01
rs4873492	T	-0.110	0.064	8.67E-02	-0.276	0.056	8.15E-07	-0.028	0.019	1.37E-01
rs488834	C	0.328	0.061	9.75E-08	0.181	0.053	7.32E-04	0.019	0.018	2.82E-01
rs4899396	T	-0.444	0.081	4.77E-08	-0.057	0.071	4.27E-01	-0.007	0.024	7.63E-01
rs4923914	T	-0.217	0.093	1.91E-02	-0.360	0.080	7.70E-06	-0.027	0.027	3.24E-01
rs507666	A	0.452	0.060	7.43E-14	0.520	0.052	3.69E-23	0.016	0.018	3.63E-01
rs5418	G	-0.236	0.071	9.04E-04	-0.206	0.062	8.84E-04	-0.014	0.021	4.92E-01
rs55646464	T	-0.405	0.105	1.13E-04	-0.485	0.090	6.50E-08	0.024	0.030	4.28E-01
rs55733296	A	-0.126	0.067	6.08E-02	-0.378	0.058	6.87E-11	-0.022	0.020	2.69E-01
rs56179563	A	-0.286	0.059	1.58E-06	-0.190	0.052	2.60E-04	-0.020	0.018	2.53E-01
rs56313611	T	0.187	0.060	1.94E-03	0.250	0.052	1.85E-06	0.012	0.018	4.92E-01
rs56352102	T	-0.153	0.070	2.89E-02	-0.265	0.061	1.32E-05	-0.048	0.021	2.08E-02
rs569550	G	0.148	0.080	6.40E-02	0.269	0.069	1.06E-04	0.016	0.023	4.84E-01
rs57139556	G	-0.354	0.059	1.93E-09	-0.148	0.051	3.81E-03	-0.012	0.017	4.93E-01
rs57786342	A	0.062	0.071	3.78E-01	0.272	0.062	1.00E-05	0.025	0.021	2.26E-01
rs6031431	G	-0.429	0.114	1.77E-04	-0.413	0.099	3.15E-05	-0.003	0.034	9.17E-01
rs604723	T	-0.349	0.059	2.81E-09	-0.360	0.051	1.82E-12	0.003	0.017	8.65E-01
rs6108171	T	-0.160	0.059	6.65E-03	-0.241	0.051	2.74E-06	-0.011	0.017	5.26E-01
rs6108787	G	0.049	0.066	4.60E-01	0.208	0.057	2.69E-04	-0.033	0.019	8.55E-02
rs62036942	C	-0.291	0.064	5.72E-06	-0.170	0.056	2.40E-03	-0.059	0.019	1.98E-03
rs62390667	G	0.241	0.066	2.71E-04	0.288	0.058	5.55E-07	0.034	0.019	7.55E-02
rs62447769	A	-0.094	0.110	3.95E-01	-0.538	0.095	1.58E-08	-0.049	0.033	1.37E-01
rs6433891	G	-0.118	0.061	5.25E-02	-0.295	0.053	2.65E-08	-0.019	0.018	2.77E-01
rs6438857	C	-0.270	0.065	3.00E-05	-0.321	0.056	1.20E-08	0.006	0.019	7.60E-01
rs645040	G	-0.139	0.064	2.98E-02	-0.363	0.055	5.70E-11	-0.031	0.019	9.56E-02
rs6490019	A	0.225	0.063	3.70E-04	0.260	0.055	2.32E-06	0.007	0.018	7.02E-01
rs66682451	G	-0.050	0.068	4.62E-01	-0.267	0.059	6.67E-06	-0.003	0.020	8.96E-01
rs6697193	T	0.086	0.059	1.45E-01	0.187	0.051	2.67E-04	0.016	0.017	3.40E-01
rs6777317	A	0.799	0.065	7.11E-35	0.550	0.056	1.21E-22	0.063	0.019	8.00E-04
rs6798940	A	0.328	0.085	1.07E-04	0.335	0.074	5.23E-06	0.027	0.025	2.79E-01
rs6815273	A	-0.874	0.201	1.33E-05	-0.745	0.168	9.46E-06	-0.065	0.060	2.76E-01
rs6827655	G	-0.494	0.113	1.26E-05	-0.521	0.096	6.69E-08	0.004	0.033	8.95E-01
rs6928622	C	-0.107	0.060	7.50E-02	-0.275	0.052	1.27E-07	-0.017	0.017	3.30E-01
rs6961048	G	0.214	0.060	3.46E-04	0.172	0.052	9.72E-04	0.029	0.017	9.59E-02
rs6963105	A	0.090	0.066	1.69E-01	0.185	0.057	1.24E-03	0.014	0.019	4.57E-01
rs6990531	G	-0.321	0.059	4.88E-08	-0.158	0.051	2.01E-03	-0.025	0.017	1.42E-01
rs7107356	A	-0.272	0.061	9.15E-06	-0.360	0.053	1.05E-11	-0.040	0.018	2.45E-02
rs7116280	C	0.440	0.085	2.52E-07	0.365	0.074	9.21E-07	0.008	0.025	7.48E-01
rs7121365	A	0.205	0.060	6.25E-04	0.162	0.052	1.85E-03	0.010	0.017	5.53E-01
rs71326977	A	0.210	0.059	3.40E-04	0.279	0.051	4.76E-08	0.020	0.017	2.56E-01
rs71508634	T	0.296	0.060	7.60E-07	0.075	0.052	1.50E-01	0.027	0.017	1.14E-01
rs71654213	T	-0.220	0.077	4.35E-03	-0.258	0.066	9.94E-05	-0.041	0.023	7.20E-02
rs7223364	T	-0.111	0.078	1.51E-01	-0.188	0.068	5.50E-03	-0.003	0.023	9.08E-01
rs72654647	A	-0.093	0.059	1.14E-01	-0.276	0.051	7.47E-08	0.004	0.017	8.31E-01
rs72677850	A	0.470	0.087	5.49E-08	0.253	0.076	7.97E-04	0.013	0.025	6.00E-01
rs72792829	T	-0.096	0.068	1.54E-01	-0.224	0.059	1.38E-04	-0.039	0.020	4.95E-02
rs72811742	T	0.228	0.061	1.65E-04	0.294	0.053	2.24E-08	0.020	0.018	2.62E-01
rs72831343	G	-0.390	0.061	1.42E-10	-0.316	0.053	2.17E-09	-0.007	0.018	7.06E-01
rs72844588	A	-0.198	0.059	7.85E-04	-0.269	0.051	1.63E-07	-0.040	0.017	1.95E-02
rs72854462	G	-0.213	0.064	8.67E-04	-0.259	0.056	3.45E-06	-0.038	0.019	4.50E-02
rs72936986	C	-0.474	0.060	2.08E-15	-0.469	0.052	2.51E-19	0.000	0.017	9.77E-01
rs72976751	T	0.339	0.073	3.66E-06	0.116	0.064	6.86E-02	0.022	0.021	3.09E-01
rs7302981	A	0.005	0.071	9.48E-01	-0.197	0.062	1.40E-03	0.017	0.021	4.18E-01
rs73030267	C	-0.131	0.059	2.59E-02	-0.183	0.051	3.50E-04	0.008	0.017	6.30E-01
rs73033340	G	0.221	0.059	1.69E-04	0.294	0.051	8.22E-09	0.031	0.017	7.34E-02

rs73046792	A	0.278	0.061	4.48E-06	0.297	0.053	1.80E-08	0.030	0.018	8.61E-02
rs7306710	T	-0.150	0.060	1.26E-02	-0.300	0.052	1.07E-08	-0.012	0.018	5.10E-01
rs73075659	G	0.065	0.059	2.68E-01	0.191	0.051	1.92E-04	0.008	0.017	6.36E-01
rs7310615	C	0.374	0.059	2.55E-10	0.465	0.051	1.28E-19	-0.021	0.017	2.36E-01
rs73234219	T	-0.677	0.114	2.84E-09	-0.611	0.099	7.78E-10	-0.048	0.034	1.57E-01
rs73306860	A	-0.256	0.118	2.92E-02	-0.568	0.103	3.94E-08	-0.063	0.035	7.23E-02
rs7340705	C	0.207	0.059	4.49E-04	0.213	0.051	3.42E-05	-0.001	0.017	9.31E-01
rs74233809	C	0.475	0.082	6.53E-09	0.400	0.071	1.82E-08	-0.018	0.024	4.41E-01
rs743509	C	-0.339	0.063	5.83E-08	-0.247	0.055	5.92E-06	-0.023	0.018	2.15E-01
rs7463212	T	0.213	0.059	3.19E-04	0.250	0.051	1.15E-06	-0.008	0.017	6.30E-01
rs74661587	G	-0.610	0.168	2.77E-04	-0.803	0.146	4.02E-08	0.030	0.049	5.38E-01
rs747423	T	0.177	0.061	3.51E-03	0.267	0.053	3.87E-07	0.020	0.018	2.48E-01
rs7484151	A	0.331	0.130	1.12E-02	0.428	0.114	1.80E-04	0.030	0.038	4.31E-01
rs748676	A	-0.276	0.059	3.17E-06	-0.220	0.051	1.86E-05	0.009	0.017	6.00E-01
rs751984	C	-0.088	0.060	1.43E-01	-0.298	0.052	1.02E-08	0.004	0.017	8.04E-01
rs76452347	T	0.252	0.078	1.30E-03	0.255	0.068	1.71E-04	0.025	0.023	2.65E-01
rs7675258	G	-0.310	0.061	3.50E-07	-0.152	0.053	4.01E-03	-0.030	0.018	8.84E-02
rs7763581	G	0.270	0.060	6.59E-06	0.149	0.052	4.30E-03	0.034	0.017	5.16E-02
rs778124	A	0.616	0.109	1.68E-08	0.661	0.095	3.14E-12	0.078	0.031	1.16E-02
rs77924615	A	0.289	0.065	8.05E-06	0.304	0.056	6.95E-08	0.018	0.019	3.31E-01
rs7810386	A	-0.058	0.060	3.30E-01	-0.306	0.052	4.53E-09	-0.029	0.018	9.61E-02
rs78203196	C	0.215	0.064	8.09E-04	0.289	0.056	1.89E-07	0.040	0.019	3.37E-02
rs7831859	C	-0.245	0.114	3.13E-02	-0.717	0.097	1.58E-13	-0.008	0.033	8.08E-01
rs7838131	G	0.174	0.098	7.61E-02	0.422	0.085	7.48E-07	0.016	0.028	5.82E-01
rs7845053	A	0.887	0.174	3.30E-07	0.224	0.152	1.40E-01	0.069	0.050	1.67E-01
rs79023617	T	-0.275	0.069	6.58E-05	-0.211	0.060	4.23E-04	-0.022	0.020	2.73E-01
rs7911644	T	-0.229	0.059	1.15E-04	-0.206	0.052	6.94E-05	0.008	0.017	6.50E-01
rs7938856	C	-0.220	0.098	2.54E-02	-0.443	0.085	2.16E-07	-0.030	0.029	2.99E-01
rs79780963	T	-0.266	0.060	9.65E-06	0.033	0.052	5.31E-01	-0.025	0.018	1.57E-01
rs7989142	G	0.137	0.059	2.06E-02	0.289	0.051	1.60E-08	0.018	0.017	2.88E-01
rs79997166	C	-0.588	0.133	1.02E-05	-0.203	0.117	8.39E-02	0.012	0.039	7.65E-01
rs8044992	C	0.299	0.060	5.00E-07	0.311	0.052	1.91E-09	0.005	0.017	7.72E-01
rs8059962	T	-0.186	0.060	1.94E-03	-0.249	0.052	1.67E-06	-0.029	0.018	1.01E-01
rs8070460	C	0.090	0.069	1.93E-01	0.286	0.060	1.86E-06	-0.011	0.020	5.79E-01
rs8112983	T	-0.303	0.068	7.78E-06	-0.239	0.059	5.37E-05	-0.014	0.020	4.95E-01
rs897264	T	0.263	0.079	8.75E-04	0.339	0.068	7.07E-07	0.001	0.023	9.54E-01
rs9314268	C	-0.143	0.076	5.84E-02	-0.211	0.065	1.26E-03	-0.022	0.022	3.19E-01
rs9375463	C	0.784	0.173	5.97E-06	0.705	0.150	2.70E-06	0.050	0.050	3.14E-01
rs9394951	C	-0.284	0.059	1.64E-06	-0.055	0.052	2.88E-01	0.005	0.017	7.64E-01
rs9482120	C	-0.138	0.059	1.98E-02	-0.149	0.052	4.00E-03	-0.002	0.017	9.26E-01
rs966791	T	-0.111	0.076	1.41E-01	-0.166	0.066	1.15E-02	0.011	0.022	6.04E-01
rs9719973	A	-0.624	0.111	1.80E-08	-0.403	0.097	2.94E-05	-0.071	0.033	3.19E-02
rs990902	C	-0.280	0.064	1.12E-05	-0.112	0.055	4.40E-02	-0.011	0.019	5.62E-01
rs9927137	G	-0.140	0.076	6.33E-02	-0.370	0.065	1.55E-08	0.009	0.022	6.75E-01
